# Genome-wide identification and characterization of *Puccinia striiformis*-responsive lncRNAs in *Triticum aestivum*

**DOI:** 10.3389/fpls.2023.1120898

**Published:** 2023-08-15

**Authors:** Parinita Das, Monendra Grover, Dwijesh Chandra Mishra, Sayanti Guha Majumdar, Bharti Shree, Sundeep Kumar, Zahoor Ahmad Mir, Krishna Kumar Chaturvedi, Subhash Chander Bhardwaj, Amit Kumar Singh, Anil Rai

**Affiliations:** ^1^ ICAR-Indian Agricultural Statistics Research Institute, New Delhi, India; ^2^ ICAR-National Bureau of Plant Genetic Resources, New Delhi, India; ^3^ ICAR-Indian Institute of Wheat and Barley Research, Shimla, Himachal Pradesh, India

**Keywords:** wheat, stripe rust, lncRNA, miRNA, mRNA

## Abstract

Wheat stripe rust (yellow rust) caused by *Puccinia striiformis* f. sp. tritici (*Pst*) is a serious biotic stress factor limiting wheat production worldwide. Emerging evidence demonstrates that long non-coding RNAs (lncRNAs) participate in various developmental processes in plants via post-transcription regulation. In this study, RNA sequencing (RNA-seq) was performed on a pair of near-isogenic lines—rust resistance line FLW29 and rust susceptible line PBW343—which differed only in the rust susceptibility trait. A total of 6,807 lncRNA transcripts were identified using bioinformatics analyses, among which 10 lncRNAs were found to be differentially expressed between resistance and susceptible lines. In order to find the target genes of the identified lncRNAs, their interactions with wheat microRNA (miRNAs) were predicted. A total of 199 lncRNAs showed interactions with 65 miRNAs, which further target 757 distinct mRNA transcripts. Moreover, detailed functional annotations of the target genes were used to identify the candidate genes, pathways, domains, families, and transcription factors that may be related to stripe rust resistance response in wheat plants. The NAC domain protein, disease resistance proteins RPP13 and RPM1, At1g58400, monodehydroascorbate reductase, NBS-LRR-like protein, rust resistance kinase Lr10-like, LRR receptor, serine/threonine-protein kinase, and cysteine proteinase are among the identified targets that are crucial for wheat stripe rust resistance. Semiquantitative PCR analysis of some of the differentially expressed lncRNAs revealed variations in expression profiles of two lncRNAs between the *Pst*-resistant and *Pst*-susceptible genotypes at least under one condition. Additionally, simple sequence repeats (SSRs) were also identified from wheat lncRNA sequences, which may be very useful for conducting targeted gene mapping studies of stripe rust resistance in wheat. These findings improved our understanding of the molecular mechanism responsible for the stripe rust disease that can be further utilized to develop wheat varieties with durable resistance to this disease.

## 1 Introduction

Wheat (*Triticum aestivum* L.) is the most widely grown crop and a major staple food crop across the world (Prasad et al., 2019). *Puccinia striiformis* f. sp. tritici (*Pst*), which causes stripe or yellow rust in wheat, is a major disease in many of the world’s wheat-growing countries. In recent decades, severe yield losses have been observed all over the world as a result of the pathogen’s rapid development and the establishment of more virulent races (Chen, 2005). Therefore, growing resistant cultivars is considered the most effective, economical, and environmentally friendly method of preventing stripe rust in wheat (Dodds and Rathjen, 2010). Plants have developed sophisticated defense systems to halt or delay the growth of pathogens in response to pathogen attacks (Dangl and Jones, 2001; Chisholm et al., 2006). A variety of defense responses, primarily the creation of reactive oxygen species (ROS), papilla formation and cell wall apposition regulated by various molecular pathways are involved in all-stage resistance against *Pst* infection in wheat (Jie, 2003; Wang et al., 2007). When subjected to stripe rust infection, wheat plants employ a precise mechanism to fight themselves from ROS attack by an efficient antioxidant defense system that includes antioxidant enzymes and antioxidant metabolites (Sairam et al., 2002; Chen et al., 2015).

Genes involved in plant defense mechanisms could be categorized into two main types: disease-resistance (R) genes and disease-resistant related genes (Dangl and Jones, 2001). A number of R genes have been reported in defense mechanisms against stripe rust, such as TaHsp90 (Wang et al., 2015), TaIF2 homolog (Zhang et al., 2013b), NGR1 encoding NB-LRR type R protein (Peart et al., 2005) and β-1,3-glucanase (Liu et al., 2010). Moreover, many other genes are differentially expressed in response to stripe rust infection (Mir et al., 2023). Therefore, profiling the transcript alterations associated with the defense response can help identify the genes and pathways affected by the pathogen infection (Eulgem et al., 2004). RNA sequencing (RNA-seq) is a comprehensive and highly effective method for analyzing the transcriptome (Shendure, 2008). In recent years, several transcriptome studies have been reported to study the underlying mechanisms involved in wheat–pathogen interactions in response to stripe rust infections in wheat (Coram et al., 2008; Yu et al., 2010; Zhang et al., 2011; Zhang et al., 2014a; Dobon et al., 2016; Yadav et al., 2016).

Long non-coding RNAs (lncRNAs) are a subclass of non-coding RNAs having more than 200 nucleotides (Kim and Sung, 2012) and are essential for various cellular processes, including transcription, post-translational processing, chromatin modification, gene expression regulation, and imprinting (Isin and Dalay, 2015). LncRNAs are involved in the regulation of downstream target gene expression via various molecular processes at transcription and post-transcription levels (Wang et al., 2018b). Although lncRNAs have a limited ability to encode proteins, they do play a function in controlling the expression of target genes during the transcription and translation processes. Recently, lncRNAs were discovered in different plant species, and they were reported to play significant roles in gene silencing (Bardou et al., 2014; Matzke and Mosher, 2014), plant growth and control of flowering time (Berry and Dean, 2015; Kim and Sung, 2017; Wang et al., 2017), organ development (Li et al., 2016), photo-morphogenesis in seedlings (Wang et al., 2014), reproduction (Zhang et al., 2014c), cell differentiation (Liu et al., 2019), and aroma formation (Varshney et al., 2019). The entire genomes and transcriptomes have been sequenced in numerous plant species, including *Arabidopsis thaliana* (Liu et al., 2012), *Oryza sativa* (Zhang et al., 2014c), *Zea mays* (Li et al., 2014b; Zhang et al., 2014b), *Cucumis sativus* (Hao et al., 2015), and *Brassica rapa* (Wang et al., 2019a), resulting in the discovery of thousands of lncRNAs. MicroRNAs (miRNAs), another important class of ncRNAs, are small RNAs of 20–22 nt in length and are involved in the regulation of gene expression at both transcriptional and post-transcriptional levels in plants (Chen, 2012). Previous research has suggested that lncRNAs can act as molecular decoys, sequestering miRNAs and, consequently, inhibiting their interaction with their target messenger RNAs (Mercer et al., 2009; Wang and Chang, 2011). Thus, lncRNAs regulate a wide range of biological processes through their interaction with miRNAs that, in turn, regulate mRNAs (Dhanoa et al., 2018). These target predictions are made more difficult by the lack of knowledge about the interfaces between lncRNAs and possible targets; however, data from genome targeting and high-throughput screening strongly suggest that lncRNAs play crucial biological functions in stress tolerance (Heo and Sung, 2011; Budak et al., 2020; Gao et al., 2020). After detecting a stress signal, PAMP-triggered immunity (PTI) is triggered by the creation of signaling molecules such as ROS. Once the virulence factors of the pathogen penetrate the plant cells, NB-LRR resistance (R) genes activate pathogen-specific effector-triggered immunity (ETI). PTI and ETI both lead to the activation of defense-related pathways. LncRNAs perform crucial regulatory roles in a number of plant defense mechanisms, by serving as either miRNA precursors or miRNA target mimics. Serval studies have revealed the role of non-coding RNAs in enhancing the biotic stress tolerance in plants and modulating the gene expression in different plant pathogen infections such as powdery mildew infection in wheat, white mold disease in rapeseed, soft rot and stem rot in potato, *Fusarium oxysporum* infection in ‘Cavendish’ banana, and eumusae leaf spot disease in banana (Xin et al., 2011; Joshi et al., 2016; Kwenda et al., 2016; Li et al., 2017; Wang et al., 2018c; Wang et al., 2018d; Borgognone et al., 2019; Kang et al., 2019; Muthusamy et al., 2019; Xing et al., 2019; Zhang et al., 2019; Zhou et al., 2019). However, very few studies have been carried out to unveil the potential regulatory role of lncRNAs and to develop the lncRNA–miRNA–mRNA network for understanding the molecular mechanism mediating stripe rust resistance in wheat (Budak et al., 2020).

We, therefore, undertook this study with the aim of using RNA-seq to identify stripe rust-associated lncRNAs in a pair of wheat near-isogenic lines (NILs), which are identical in their agricultural traits except for a substantial difference in disease response. We aimed to identify common stripe rust disease-associated lncRNAs in the NILs to obtain candidate lncRNAs and their potential regulatory targets for functional study and further high-yield variety breeding. Studying the role of lncRNAs during biotic stress conditions will be vital to engineering plants for durable stress tolerance.

## 2 Materials and methods

### 2.1 Plant material and collection of samples

A NIL FLW29 containing yellow rust resistance gene *Yr16* introgressed from a wheat variety ‘Cappelle-Desprez’ was crossed with wheat cultivar PBW343. The recipient parent FLW29 (resistant) and cultivar PBW343 (susceptible) were used for identifying the lncRNAs in response to *Pst* infection. Two wheat genotypes were inoculated with *Pst* pathotype 46S119 such that for each cultivar a total of 10 pots (five mock-inoculated and five inoculated) with three biological replicates at three different time durations (12, 48, and 72 hpi) were used for the experiment. Seedlings of both the cultivar were grown in plastic pots (20 × 20 × 20 cm^3^) at a distance of 1.5 cm (seed to seed) at the Indian Council of Agriculture Research-Indian Institute of Wheat Barley and Research (ICAR-IIWBR), Regional Station, Flowerdale, Shimla. When the plants attained the two-leaf stage (~15 days after sowing), fresh urediniospores of pathotype 46S119 were harvested from the infected wheat plants and suspended in sterile distilled water containing Soltrol (20 mg/100 ml), which helps in pathogen adherence to leaves. The spore suspension was sprayed on seedlings using an atomizer and later kept in the dark at 10°C for 16-h light/8-h dark to maintain the relative high humidity. Leaf samples were collected at three different time periods, i.e., at 12, 48, and 72 h post-inoculation. The collected samples were immediately placed in RNAlater^®^ and stored at −20°C until use. Four samples each at three time points with a total of 12 sample combinations, viz., resistant inoculated (FLW_T12, FLW_T48, and FLW_T72), resistant control (FLW_C12, FLW_C48, and FLW_C72), susceptible inoculated (PBW_T12, PBW_T48, and PBW_T72), and susceptible control (PBW_C12, PBW_C48, and PBW_C72), each with three biological replications, were pooled to increase the detection accuracy of transcriptome analysis.

### 2.2 RNA extraction, library construction, and sequencing

Total RNA was isolated from *Pst*-treated and mock-inoculated leaf samples using the Qiagen RNeasy Mini kit (Qiagen Inc., Valencia, CA, USA) according to the manufacturer’s instructions, including the recommended treatment with DNase. The RNA quality was verified using RNA 6000 Nano Kit (Agilent Technologies, Santa Clara, CA, USA) on 2100 Bioanalyzer (Agilent Technologies, USA). RNA concentrations were determined with a NanoDrop ND-8000 spectrophotometer (NanoDrop Technologies, Wilmington, DE, USA; Thermo Scientific, Wilmington, DE, USA). cDNA libraries were constructed using an Illumina-TrueSeq RNA library preparation kit (Illumina Inc., San Diego, CA, USA) according to the manufacturer’s recommended protocol and sequencing was carried out on single HiSeq 4000 lane using 150-bp paired-end chemistry. The library preparation and sequencing were performed by commercial service providers (NxGenBio Life Sciences, New Delhi, India). Briefly, total RNA was used to purify poly(A) messenger RNA (mRNA) using oligo-dT beads to capture polyA tails and attached magnetic beads were used for two rounds of purification. During the second elution, enriched mRNA was fragmented into 200–500-bp pieces using divalent cations at an elevated temperature (94°C) for 5 min. With the use of SuperScriptII reverse transcriptase (Life Technologies, Inc., Carlsbad, CA, USA) and random primers, the cleaved RNA fragments were transcribed into first-strand cDNA. Fragments were end-repaired and A-tailed after second-strand cDNA synthesis and indexed adapters were ligated. To construct the final cDNA library, the products were purified and enriched by PCR. Libraries were sequenced using the paired-end (100 bp at each end) module of the Illumina HiSeq platform. After sequencing, the samples were demultiplexed and the indexed adapter sequences were trimmed using the CASAVA v1.8.2 software (Illumina Inc.).

### 2.3 Bioinformatics pipeline for identifying lncRNAs

**Figure 1:**
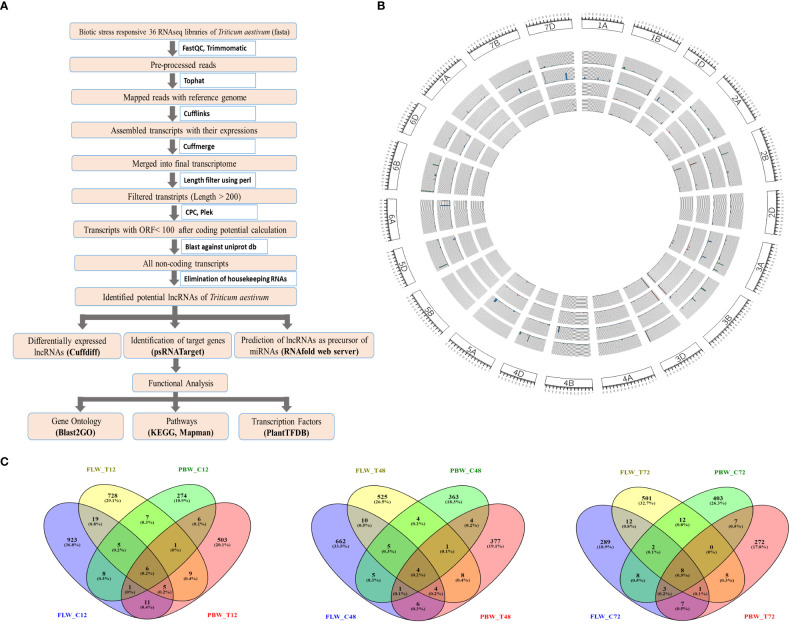
Identification and characterization of lncRNAs in wheat. **(A)** The pipeline for the identification of lncRNAs in wheat. **(B)** Circos plot depicting the distribution and expression of identified lncRNAs. From outer to inner circles, lncRNA distribution is represented on chromosomes with their expression levels (FPKM value) of lncRNAs in the samples of FLW29 control, PBW343 control, FLW29 treated, and PWB343 treated. **(C)** Venn diagram representing number of unique and shared lncRNAs between FLW29 and PBW343 under control and *Pst*-treated conditions from 12-, 48-, and 72-h samples. lncRNAs, long non-coding RNAs; FPKM, fragments per kilo of transcript per million reads mapped.

The bioinformatics pipeline that was followed to identify the *T. aestivum* lncRNA transcripts is depicted in **Figure 1**. Each fastq file was aligned with the *T. aestivum* reference genome (RefSeqv1.0; IWGSC, 2018) using Tophat (Trapnell et al., 2009) and the Cufflinks package was used to build the aligned reads (Trapnell et al., 2010). Cuffmerge was used to merge all transcript files into a single non-redundant transcriptome (Trapnell et al., 2010). The FASTA file was extracted from the combined GTF file using the GFF reads module of the Cufflinks package. Transcripts having lengths of <200 bp were removed by length filters using Bioperl code. These filtered transcripts were assessed using Coding Potential Calculator2 (CPC2) (Kang et al., 2017) and PLEK (predictor of long non-coding RNAs and messenger RNAs based on an improved k-mer scheme) (Li et al., 2014a) to calculate the coding potential, and only non-coding transcripts were retained for further analysis. The obtained transcripts were searched against tRNA, rRNA, snRNA, and snoRNA databases to filter out housekeeping genes using BLASTN. Further, in order to eliminate the coding transcripts, BLASTX was performed against the wheat protein downloaded from the UniProt database.

### 2.4 Differential expression analysis of the lncRNAs

Identified lncRNAs were checked for their differential expression pattern between NILs under control and *Pst*-infected plants at three different time points. The calculation of values of expression in the form of fragments per kilo of transcript per million reads mapped (FPKM) was performed using the Cufflinks tool. Further, these expression values were compared between PBW343 and FLW29 lines for both control and treated conditions at each time point by the Cuffdiff tool (Trapnell et al., 2012) in the form of log(fold change) value. The lncRNAs that met the criteria of a threshold value of absolute log_2_ fold change ≥2 (upregulated) and ≤−2 (downregulated) and p-value ≤0.001 were considered as differentially expressed lncRNAs.

### 2.5 Identification of SSR-bearing lncRNAs

Simple sequence repeats (SSRs) are microsatellite markers that are crucial for molecular characterization and provide useful insights into plant genetic diversity (Misganaw and Abera, 2017). They are co-dominant, highly variable, and uniformly dispersed over the whole genome (Pinto et al., 2006). For the identified putative lncRNA, the Krait tool (Du et al., 2018) was used to determine the frequency and distribution of SSRs (mono, di, tri, tetra, penta, and hexa). The default parameters of the Krait tool with respect to frequency of repeats were employed for predicting SSRs, viz., 10, 7, 5, 4, 4, and 43 for mono, di, tri, tetra, penta, and hexa nucleotide repeats, respectively.

### 2.6 Prediction of lncRNAs as a precursor of miRNAs

To identify the lncRNA functions as a precursor of miRNA, the 122 precursor sequences of known 119 miRNAs were downloaded from the miRBase database (http://microrna.sanger.ac.uk/) (Griffiths-Jones et al., 2006, 2008) and aligned with identified lncRNAs. A lncRNA harboring a miRNA precursor sequence with 100% query coverage and similarity was considered a precursor of that miRNA. The hairpin loop formation in lncRNAs was analyzed using the miRNAFold server (https://evryrna.ibisc.univ-evry.fr/miRNAFold) (Tav et al., 2016), and the secondary structure was plotted using the Vienna RNAfold web server (http://rna.tbi.univie.ac.at/) (Gruber et al., 2015).

### 2.7 Interaction of lncRNAs with miRNAs and mRNAs

A total of 119 previously reported mature miRNAs of *T. aestivum* downloaded from the miRBase database (http://microrna.sanger.ac.uk/) (Griffiths-Jones et al., 2008) were used for interaction analyses with lncRNAs using psRNATarget (https://www.zhaolab.org/psRNATarget//) (Dai and Zhao, 2011). The lncRNAs that showed interaction with miRNAs were then considered for interaction analyses with mRNAs (coding sequence or CDS) of *T. aestivum* downloaded from National Center for Biotechnology Information (NCBI)) with an expected value threshold of 3.0 and target accessibility set at a maximum of 25. The interaction network of lncRNAs, miRNAs, and mRNAs was visualized using Cytoscape (http://cytoscapeweb.cytoscape.org/) (Shannon et al., 2003) version 3.9.1.

### 2.8 Analysis of GO and KEGG pathways of target genes of lncRNA

To understand the functional characteristics of the target genes of the identified lncRNAs, blastx was performed against the nr database. This was performed to identify the proteins that had the highest sequence similarity with the given transcripts to retrieve their functional annotations, and a typical cutoff e-value < e−10 was set. BLAST2GO (http://www.blast2go.com/b2ghome) (Conesa et al., 2005) program was used to obtain Gene Ontology (GO) annotations of the differentially expressed lncRNAs (DELs) for describing biological processes, molecular functions, and cellular components. The GO graph of the targeted genes of the identified lncRNAs was generated using the WEGO program (Ye et al., 2018). The Kyoto Encyclopedia of Genes and Genomes (KEGG) analysis was performed on the KOBAS website (Bu et al., 2021).

### 2.9 Comparative analysis of *T. aestivum* lncRNAs with other plant species

To find the homology of *T. aestivum* lncRNAs with the previously known and reported lncRNA sequences of other plant species, BLASTn analysis was performed using an e-value of 0.001. Other known lncRNAs of different plant species were downloaded from different lncRNA databases. LncRNAs of *Hordium vulgare* were downloaded from CANTATAdb (http://cantata.amu.edu.pl/index.html), while *O. sativa*, *Zea mays*, and *Sorghum bicolor* were downloaded from GREENC database (http://greenc.sciencedesigners.com/). Also, to check the homology of the identified lncRNA to the wheat CDS, BLASTn was performed with percent identity >85%, query coverage >80%, and e-value < e^−50^.

### 2.10 Expression analysis of lncRNAs using semiquantitative PCR

Wet lab expression analysis of a few *Pst*-induced differentially expressed lncRNAs was performed using semiquantitative PCR. For this analysis, total RNA from *Pst*-treated and mock-inoculated leaf samples was isolated at two time points (12 and 72 hpi) following the procedure described in the section, and it was purified using TURBO DNA-free™ Kit (Thermo Fisher Scientific, Waltham, MA, USA) to eliminate any chances of genomic DNA contamination. High-quality RNA was used to synthesize cDNA using the Revert Aid First Strand cDNA Synthesis Kit (Thermo Fisher Scientific, USA) according to the manufacturer’s instructions (Thermo Scientific, USA). Primer3 program was used to design gene-specific primers for a total of eight lncRNAs that were found to be differentially expressed between rust susceptible and resistant lines using RNA-seq analysis (**Table S15**). Additionally, a wheat actin gene segment was amplified as a positive control using the primer pair 5′ CCAAGGGCTGTTTTCCCTAG 3′ and 5′ CTCAAGTACCCGATTGAGCA 3′. Amplification reactions were set up in 20-μl volume containing, 1 U of *Taq* DNA polymerase, 1× PCR buffer, 250 mM of dNTPs, 100 ng of cDNA, and primers at the concentration of 0.2 μM. The PCR program was as follows: 95°C for 5 min, followed by 35 cycles each consisting of 94°C for 1 min, 55°C–58°C for 1 min and 72°C for 1.5 min, and finally at 72°C for 8 min. Amplified products were separated on 2.0% agarose gel at constant 100 V with 1× Tris acetate EDTA (TAE) buffer (pH −8.0) and visualized in a gel documentation system (AlphaImager, American Instrument Exchange, Haverhill, MA, USA).

## 3 Result

### 3.1 Identification of putative lncRNAs

The total assembled 164,095 transcripts generated from Cuffmerge was filtered by length using a perl script where 340 transcripts having lengths less than 200 nt were discarded, and a total of 163,755 transcripts were retained. These transcripts were assessed using the CPC tool based on open reading frame (ORF) integrity and p-value. All coding labeled parts were filtered, which had ORF lengths less than 100 and p-value less than 0.5, and after that, 40,316 transcripts were obtained. Likewise, the Plek tool was also used to find out the non-coding RNAs. After filtering the coding RNAs, the remaining 57,200 transcripts were classified as non-coding. Further, the common transcripts IDs from CPC and PLEK results were obtained, and a total of 27,628 transcripts IDs were found to be common in both CPC and PLEK filters. By performing BLASTn against the non-coding RNA databases downloaded from the RNA Central (https://rnacentral.org/), genes like rRNA, tRNA, snoRNA, and snRNA were removed from the remaining transcripts. There were 359, 26, 55, and 2 hits found against rRNA, tRNA, snoRNA, and snRNA databases, respectively. After gradually removing all these hits, 27,186 transcripts were retained. Further, the BLASTX program was run against the UniProt database to remove protein parts from these reads. As a result of BLASTX, 7,031 hits were found and discarded, and a total of 20,155 transcripts were obtained. These transcripts were further filtered for their exon count level, and 13,347 mono-exonic transcripts were removed from subsequent analysis. The remaining 6,807 transcripts having more than one exon were identified as the putative lncRNAs. A total of 977, 696, 329,779, 560, and 540 lncRNAs were identified from samples FLW_C12, FLW_C48, FLW_C72, FLW_T12, FLW_T48, and FLW_T72, respectively. Similarly, in the case of PBW343, a total of 307, 386, 442, 541, 404, and 302 lncRNAs were identified from samples PBW_C12, PBW_C48, PBW_C72, PBW_T12, PBW_T48, and PBW_T72, respectively (**Table 1**). It was observed that 923 (36.8%), 728 (29.1%), 274 (10.9%), and 503 (20.1%) lncRNAs were uniquely present under FLW control, FLW-treated, PBW control, and PBW-treated conditions, respectively, at 12 hpi. Further, 6 (0.2%) lncRNAs were found to be common in all the conditions at 12 hpi. At 48 hpi, 662 (33.5%), 525 (26.5%), 363 (18.3%), and 377 (19.1%) lncRNAs were unique for FLW control, FLW-treated, PBW control, and PBW-treated conditions, respectively. There were four (0.2%) lncRNAs common in all the conditions at 48 hpi. Similarly, 289 (18.9%), 501 (32.7%), 403 (26.3%), and 272 (17.8%) lncRNAs were unique for FLW control, FLW-treated, PBW control, and PBW-treated conditions, respectively, at 72 hpi (**Figure 1C**). It was also observed that eight (0.5%) lncRNAs were common in all the conditions at 72 hpi. Further, comparing all the common lncRNA at 12, 48, and 72 hpi, it was observed that only four lncRNAs were common in both the lines of both control and treated samples in all the different time points, which indicates that these lncRNAs are expressed under all the conditions.

**Table 1:** List of numbers of lncRNAs in different conditions.

Lines	Conditions	12 h	48 h	72 h
FLW29	Control	977	696	329
	Treatment	779	560	540
PBW343	Control	307	386	442
	Treatment	541	404	302

### 3.2 Basic features and characterization of lncRNA transcripts

The length distribution of the lncRNAs showed that the average nucleotide length of the lncRNAs of the FLW29 line was 1007, 973, 986, 966, 938, and 926 bp for C12, T12, C48, T48, C72, and T72, respectively. In the case of PBW343, the average nucleotide length found was 911, 968, 911, 984, 874, and 902 in C12, T12, C48, T48, C72, and T72 conditions, respectively. It was observed that the lengths of lncRNAs ranged from 203 to 5428 bp, with the vast majority having lengths between 600 and 900 bp under different conditions (**Figure S1**). The identified lncRNAs of both lines showed that the exon count was between 2 and 14, and the maximum number of lncRNAs was bi-exonic followed by exon numbers 3, 4, 5, and so on (**Figure S1**). In this study, 2,765 lncRNAs had two exons, 1,616 had three exons, 1,097 had four exons, and the rest had between 5 and 14 exons. Chromosomal distribution of the identified lncRNA was visualized using the Circos software, depicted in **Figure 1B**.

### 3.3 Conservation analysis of identified wheat lncRNAs

To check the conservation level of identified wheat lncRNAs with other species, blastn of lncRNAs was performed against lncRNAs of other plant species with the parameters, e-value < e^−10^, coverage > 50%, and percent identity >35%. A total of 217 significant blast hits were found out of which 99 lncRNAs were unique. Therefore, out of 6,807 lncRNAs, 99 were found homologous with the other cereal crops. LncRNAs of *H. vulgare* were found closer to the wheat lncRNA followed by the lncRNAs of *O. sativa*, *Z. mays*, and *S. bicolor*. The first 15 hits of lncRNA were listed with percent identity, coverage, and e-value (**Table S9**).

To test the conservation of identified lncRNAs with the protein-coding genes, lncRNAs were tested for homology to wheat CDSs by BLAST with percent of identity >85%, query coverage >80%, and e-value < e^−50^. The result showed that a total of 46 lncRNAs were homologous with 178 wheat CDS, among which one lncRNA (TCONS_00009015) was found homologous to 11 CDSs related to a receptor-like kinase (RLK) or more specifically serine/threonine kinase function. The detailed information of these lncRNAs when mapped with the CDSs of bread wheat is presented in **Table S8**. This finding is consistent with the literature, which suggests that lncRNAs have very low conservation levels when compared to protein-coding mRNAs (CDS) and are species- and tissue-specific.

### 3.4 Differentially expressed lncRNAs between NILs

The FPKM values generated by Cufflinks were used further for obtaining DELs between the two lines—FLW29 and PBW343—under different conditions using the Cuffdiff software. A total of 10 significant differentially expressed lncRNAs were found, out of which five were upregulated and five were downregulated. In these five upregulated DE-lncRNAs, one lncRNA (TCONS_00163170) was common in C48, C72, T48, and T72, and four DELs were uniquely present, out of which three were from control condition (TCONS_00076516, TCONS_00093548, and TCONS_00100461) and one was from *Pst*-infected condition (TCONS_00073476). In the case of five downregulated DE-lncRNAs, one was from control conditions and four were from *Pst*-infected conditions. In these five downregulated DE-lncRNAs, all were uniquely expressed, out of which one was expressed in control (TCONS_00025410) and four in *Pst*-infected conditions (TCONS_00040012, TCONS_00053873, and TCONS_00066365) (**Table S7**).

### 3.5 LncRNAs as potential miRNA precursors and endogenous target mimics

**Figure 2:**
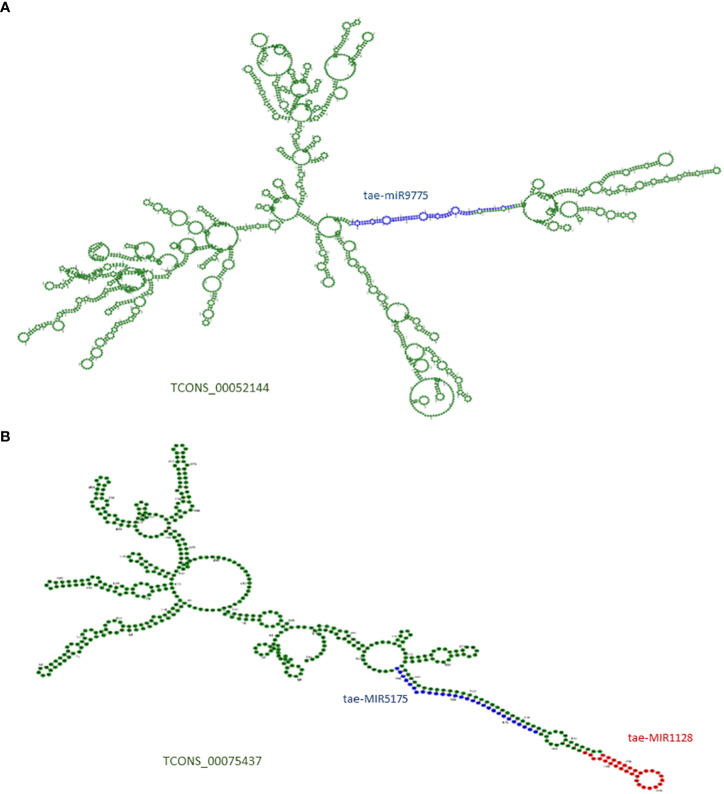
**(A)** Secondary structure of lncRNA TCONS_00052144, which acts as putative precursor of miRNA (tae-miR9775). **(B)** Secondary structure of lncRNA TCONS_00075437, which acts as putative precursor of two miRNAs (tae-miR5175 and tae-miR1128). The precursor region of miRNA are marked with blue and red; The mature miRNA regions are marked in green colors.

LncRNAs are long RNAs found in the nucleus, nucleolus, and/or cytoplasm that can serve as a precursor for smaller ncRNAs such as snRNAs, snoRNAs, and miRNAs. We explored the lncRNAs acting as precursors of known miRNAs in *T. aestivum* using the miRNAFold server. A total of 13 lncRNAs were predicted as a precursor of 10 miRNAs, which suggests that those lncRNAs can give rise to the mature miRNAs after being acted upon by nuclease enzymes like dicer and/or drosha (**Table S10**). **Figure 2** shows lncRNA TCONS_00052144 containing the precursor and mature sequences of miRNA tae-MIR9775 (**Figure 2A**) and another lncRNA, TCONS_00075437, acting as a precursor of two miRNAs: tae-MIR1128 and tae-MIR5175 (**Figure 2B**).

**Figure 3:**
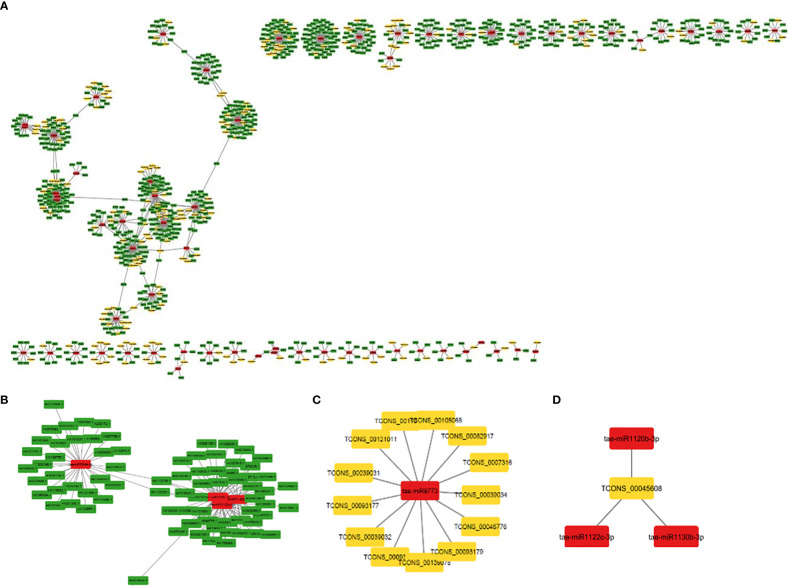
**(A)** An interaction network shows association between lncRNAs, miRNAs, and mRNAs. The yellow, red, and green nodes represent the lncRNAs, miRNAs, and mRNAs, respectively. **(B)** Interaction of a miRNA with multiple mRNAs and **(C)** lncRNAs. **(D)** Interaction of a lncRNA with multiple miRNAs.

**Figure 4:**
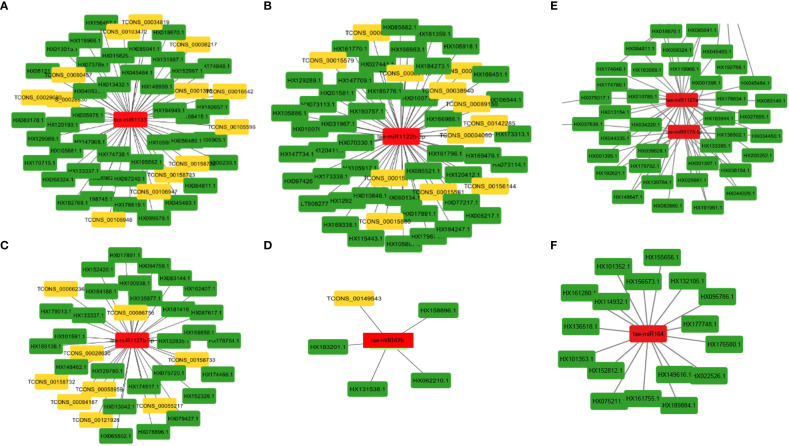
MiRNA as hub genes and their targeted lncRNAs and mRNA involved in the wheat stripe rust resistance. Gene targets of the miRNAs **(A)** tae-miR1133, **(B)** tae-miR1122b-3p, **(C)** tae-miR1127b-3p, **(D)** tae-miR167b, **(E)** tae-miR5175-5p and tae-miR1127a, and **(F)** tae-miR164.

MiRNAs are short ncRNAs (18–23 nt) that regulate mRNA expression by binding to the 3′ UTR of protein-coding mRNAs. Depending on the complementarity of the miRNA–mRNA interaction, expression is inhibited or silenced. Full complementarity causes mRNA degradation, which silences the genes, whereas partial complementarity reduces mRNA expression, which downregulates the genes. LncRNAs can sometimes interfere in this process by acting as a miRNA sponge, preventing miRNA–mRNA binding. Identified lncRNAs and known miRNAs of wheat available at the psRNAtarget server were taken for this analysis. The identified wheat lncRNAs were uploaded as target sequences against the available wheat miRNAs to the psRNAtarget server and executed with the parameters of max UPE 25 and expectation ≤ 3. A total of 233 interactions with 199 unique lncRNAs and 65 unique miRNAs of *T. aestivum* were found (**Table S11**). Target mRNAs of the identified miRNAs were also found using psRNAtarget by submitting wheat CDS as a target and previously identified 65 miRNAs as small RNAs. A total of 902 miRNA–mRNA interactions were found with 757 distinct mRNA transcripts involved in various functions. Individual lncRNA–miRNA and miRNA–mRNA networks were integrated and visualized using the Cytoscape software (**Figure 3A**), revealing an interaction network of lncRNAs, miRNAs, and mRNAs involving miRNA and target mRNAs that can possibly be interfered with by lncRNA, hence affecting the normal gene regulatory process. Multiple sets of interactions were detected such as a single lncRNA (TCON00045608) interacting with three miRNAs and a single miRNA interacting with several lncRNAs and mRNAs (**Figures 3B–D**, **Table S11**). A total of 50 interconnected clusters were present in the network. Further, hub genes were identified by using the application cytoHubba, which is itself a part of the Cytoscape tool. The top 1 hub gene was identified on the basis of 11 different algorithms, viz., MCC, DMNC, MNC, Degree, EPC, BottleNeck, EcCentricity, Closeness, Radiality, Betweenness, Stress, and Clustering Coefficient. A total of four hub genes (tae-miR1133, tae-miR1122b-3p, tae-miR1127b-3p, and tae-miR167b) were identified, and their targeted lncRNAs and mRNA are given in **Figures 4A–D**, respectively.

### 3.6 Functional analysis of the target genes of lncRNAs

**Figure 5:**
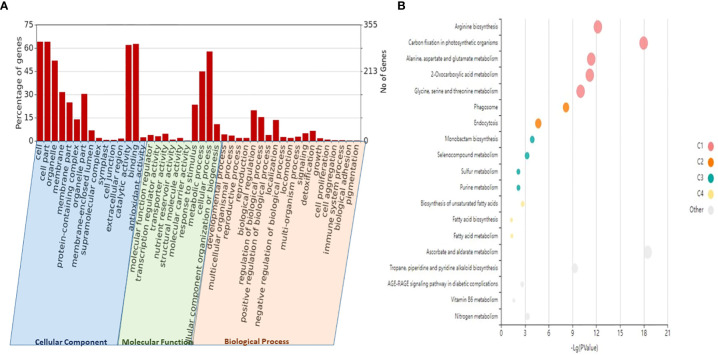
GO and KEGG enrichment analyses of the identified lncRNAs and their potential target genes. **(A)** Different biological process (BP), molecular function (MF), and cellular component **(CC)** enriched in the target genes of lncRNA plotted through WEGO (p-value <0.05). **(B)** The KEGG enrichment bubble plot (p-value <0.05) of target genes of lncRNAs. The size of the circles represents the number of genes, and the color of the circle represents the p-value. GO, Gene Ontology; KEGG, Kyoto Encyclopedia of Genes and Genomes; lncRNAs, long non-coding RNAs.

To investigate the functions of lncRNAs, we analyzed the potential targets of lncRNAs by finding target miRNAs and their interactions with known protein-coding genes or mRNAs of wheat. In our study, 757 target genes of lncRNAs were annotated, which are involved in a variety of metabolic processes like biotic stress tolerance, disease resistance, regulation of cell cycle, and cell morphogenesis. Specifically, we observed many lncRNAs targeting biotic stress response-related genes like serine/threonine-protein kinase, cytochrome P450, NBS-LRR-like protein, rust resistance kinase Lr10-like, putative disease resistance protein RPM1, RPP13, At1g58400, and transcription factors like bZIP, NAC, and MYB, which signify their roles in biotic stress response (**Table 2**). The functional annotation revealed a predominance of different GO categories for the analyzed target genes. It revealed that a total of 20 biological processes, 13 molecular functions, and two cell components were significantly altered in response to stripe rust. The most significantly enriched biological process includes the GO terms like regulation of cellular (GO:0009987), metabolic (GO:0008152), biological regulation (GO:0065007), response to stimulus (GO:0050896), localization (GO:0051179), and immune system process (GO:0002376). Among molecular functions, GO terms like binding (GO:0005488), ATP-dependent activity (GO:0140657), catalytic activity (GO:0003824), antioxidant activity (GO:0016209), and transporter activity (GO:0005215) were significantly enriched. Further, for the cellular component, the GO terms like cellular anatomical entity process (GO:0110165) and protein-containing complex (GO:0032991) were regulated (**Figure 5**, **Table S12**). With the use of the Kobas tool, the KEGG analysis revealed 27 significantly enriched pathways (p ≤ 0.05) in the targeted genes of the identified lncRNAs, some of which were related to the metabolism of ascorbate and aldarate, carbon fixation in photosynthetic organisms, metabolic pathways, arginine biosynthesis, purine metabolism, and the biosynthesis of secondary metabolites, having key significance in plants in disease response (**Figure 5**, **Table S13**). Moreover, 11 domains with significant importance to biotic stress response have been found in the targeted genes of lncRNA such as NAD-binding domain (IPR006140), serine/threonine-specific protein phosphatase (IPR006186), serine-threonine/tyrosine-protein kinase catalytic domain (IPR001245), ubiquinol–cytochrome *c* reductase hinge domain (IPR023184), NAD-dependent epimerase/dehydratase (IPR001509), reverse transcriptase zinc-binding domain (IPR026960), protein kinase domain (IPR000719), WRKY domain (IPR003657), and NAC domain (IPR003441) (**Figure S2**).

**Table 2:** Lists of lncRNA and miRNA target genes involved in the wheat stripe rust resistance.

LncRNA accession	MiRNA accession	Target mRNA accession	Target mRNA/gene description
TCONS_00079585	tae-miR164	HX177748.1, HX149616.1, HX176580.1, HX101352.1, HX101353.1, HX155656.1, HX189884.1, HX156573.1, HX022526.1, HX132105.1, HX161755.1, HX136518.1, HX114932.1, HX161280.1, HX152812.1, HX095786.1, HX075211.1, HX152812.1	NAC domain-containing protein 21/22-like; NAC domain-containing protein 92-like; putative disease resistance RPP13-like protein 3; putative disease resistance protein RPP13
TCONS_00046493, TCONS_00142862, TCONS_00142861, TCONS_00142859, TCONS_00146761, TCONS_00151741	tae-miR5175-5p	HX039628.1, HX010785.1, HX038154.1, HX027855.1, HX044329.1, HX029961.1, HX034450.1, HX044335.1, HX138502.1, HX001397.1, HX034220.1, HX001395.1, HX001396.1, HX133395.1, HX192621.1, HX178634.1, HX149647.1, HX139764.1, HX163944.1, HX191961.1	Monodehydroascorbate reductase
TCONS_00155902, TCONS_00103472, TCONS_00147277, TCONS_00147276, TCONS_00029083, TCONS_00013986	tae-miR1127a	HX083149.1, HX082660.1, HX133337.1, HX174790.1, HX037638.1, HX013154.1, HX085041.1, HX119966.1, HX200252.1, HX058324.1, HX045484.1, HX045483.1, HX075017.1, HX039628.1, HX010785.1, HX027855.1, HX038154.1, HX044329.1, HX034450.1, HX029961.1, HX044335.1, HX138502.1, HX001397.1, HX034220.1, HX001395.1, HX001396.1, HX133395.1, HX192621.1, HX178634.1, HX149647.1, HX139764.1, HX163944.1, HX191961.1, HX018670.1, HX183069.1, HX174646.1, HX192768.1, HX084811.1, HX133337.1	NAC domain-containing protein 78; NBS-LRR-like protein; peroxidase 70-like; monodehydroascorbate reductase; NBS-LRR-like protein
TCONS_00141524, TCONS_00141522, TCONS_00118627	tae-miR408	HX194411.1	SUMO-activating enzyme subunit 1A-like
TCONS_00019414, TCONS_00019413	tae-miR9778	HX106350.1, HX052028.1, HX096989.1, HX031895.1	Disease resistance protein RPP13; Disease resistance protein RPM1; putative disease resistance protein At1g58400
TCONS_00052144	tae-miR7757-5p	HX200234.1	Disease resistance protein RPM1
TCONS_00060314, TCONS_00039011, TCONS_00079707, TCONS_00106947, TCONS_00071456, TCONS_00106946, TCONS_00106946, TCONS_00155902, TCONS_00004942, TCONS_00030417, TCONS_00106945, TCONS_00110167, TCONS_00087992	tae-miR1128	HX065511.1	Rust resistance kinase Lr10-like
TCONS_00111427, TCONS_00111428, TCONS_00111425	tae-miR159a	HX061200.1	LRR receptor-like serine/threonine-protein kinase At3g47570 isoform X2
TCONS_00156144, TCONS_00089148, TCONS_00015581, TCONS_00038945, TCONS_00015576, TCONS_00065348, TCONS_00015580, TCONS_00015579, TCONS_00089150, TCONS_00038940, TCONS_00004060, TCONS_00142285	tae-miR1122b-3p	HX193757.1, HX147734.1, HX169476.1, HX129289.1, HX173338.1, HX184273.1, HX161796.1, HX166988.1, HX106544.1	Cysteine proteinase EP-B 2-like isoform X6
TCONS_00106947, TCONS_00008217, TCONS_00028630, TCONS_00029083, TCONS_00103472, TCONS_00106946, TCONS_00016542, TCONS_00105596, TCONS_00060457, TCONS_00034819, TCONS_00013986, TCONS_00158732, TCONS_00158733	tae-miR1133	HX198745.1, HX195582.1, HX170715.1, HX106415.1	Cysteine proteinase EP-B 2-like
TCONS_00156144, TCONS_00089148, TCONS_00015581, TCONS_00038945, TCONS_00015576, TCONS_00065348, TCONS_00015580, TCONS_00015579, TCONS_00089150, TCONS_00038940, TCONS_00004060, TCONS_00142285	tae-miR1122b-3p	HX184247.1, HX169451.1, HX161770.1, HX129266.1, HX173313.1, HX166963.1, HX179674.1, HX147709.1	Cysteine proteinase EP-B 2-like isoform X6

### 3.7 Identification of lncRNAs containing SSRs

All of the lncRNA transcripts found in this research were utilized to find potential microsatellites using Krait v1.1.0, a powerful and fast tool with a user-friendly graphic interface for identifying microsatellites across the genome (Du et al., 2018). A total of 635 lncRNAs were identified as having SSRs out of a total of 6,807 putative lncRNAs. The number distribution of SSRs detected from lncRNAs as mono, di, tri, tetra, penta, and hexa were 35, 180, 314, 75, 21, and 9, respectively (**Figure S3**, **Table S14**). Among the microsatellites, the di-nucleotide (CT) motif was the most frequent (8%), followed by the tri-nucleotide (CCG) pattern (5%).

### 3.8 Expression analysis using semiquantitative PCR

**Figure 6:**
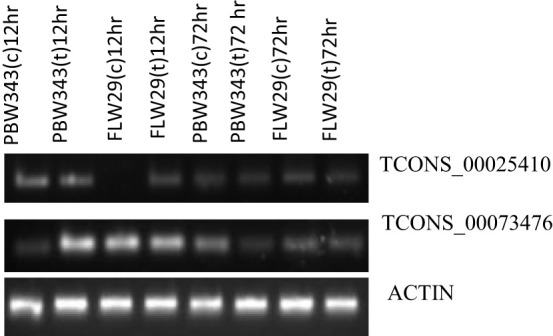
Semiquantitative PCR expression analysis of two lncRNAs in PBW343 and FLW29 at different time points. Wheat actin was used as a reference gene.

Of the eight lncRNAs that we analyzed, the expression of only two (TCONS_00025410 and TCONS_00073476) could be detected, and their expression, in general, varied between susceptible and resistant genotypes and over two time points (12 and 72 hpi), although only slightly. At 12 hpi, both mock-inoculated and *Pst*-inoculated plants of sensitive genotypes showed upregulation of the lncRNA *TCONS_00025410*. However, this lncRNA was not expressed in mock-inoculated plants but showed upregulation in *Pst*-inoculated plants at the same time point (12 hpi) in the resistant genotype. At 72 hpi, TCONS_00025410 was downregulated in both sensitive and resistant genotypes and in both mock-inoculated and *Pst*-inoculated plants. The expression of another lncRNA, TCONS_00073476, was downregulated in mock-inoculated plants of sensitive genotype at 12 hpi. However, it was upregulated in the *Pst*-inoculated plants of sensitive genotype and both the mock and *Pst*-inoculated plants of resistant genotypes at the same time point. However, at 72 hpi, this RNA showed downregulation in mock as well as *Pst*-inoculated plants in both sensitive and resistant genotypes (**Figure 6**).

## 4 Discussion

In recent years, non-coding RNAs including miRNA and lncRNAs have immerged as the master regulator of genes that are associated with the development of biotic and abiotic stress responses in plants. Plant miRNAs are important regulators that engage in regulatory functions at the post-transcriptional levels (Reinhart et al., 2002) and also play very important roles in plant defense responses (Aukerman and Sakai, 2003; Feng et al., 2015; Yu et al., 2017). The role of miRNA in plant growth and development and responses to stress is well documented; however, the role of lncRNAs in these processes is yet to be fully explored. In wheat, few lncRNAs have been identified, which play a role in abiotic stress responses, seed germination, and disease resistance (Xin et al., 2011; Zhang et al., 2013a; Shumayla et al., 2017; Zhou et al., 2020). Although progress has been made toward understanding the molecular mechanisms behind stripe rust resistance, little is known about the potential roles of lncRNAs in response to stripe rust.

The plant response to pathogen infection is highly complex at the molecular level involving one or a few major genes upstream and several minor genes downstream (Jain et al., 2020). In addition to protein-coding genes, variations in the expression of non-coding regulators such as miRNA and lncRNA also have a role in defining plant immunity against pathogens. LncRNAs work at multiple levels via simple or complex molecular mechanisms to affect gene regulation (Wang and Chang, 2011; Chekanova, 2015; Wang and Chekanova, 2017). i) LncRNAs can act in a *cis* or *trans* manner and work by complementing the sequence of RNA or DNA. ii) LncRNAs can also act as miRNA precursors at the most fundamental level. iii) LncRNAs can function as molecular sponges or decoys for miRNAs and RNA-binding proteins. They serve as decoys that prevent the access of regulatory proteins to DNA or RNA by mimicking their targets. They may also interact with miRNAs as competitors and function as miRNA target mimics or prevent microRNAs from binding with their targets.

Therefore, in this study, we attempted to identify lncRNA of wheat in response to stripe rust and discovered 6,807 lncRNA transcripts, 10 of which were found to be differentially expressed between FLW29 (resistant) and its NIL, PBW343 (susceptible). The total number of lncRNA identified in this study is comparable to that of other studies in wheat near-isogenic lines (Zhou et al., 2020). The identified wheat lncRNAs had an average length of 987 bp, which was much greater than the lengths reported for potato (895), rice (800 bp), and chickpeas (614 bp) (Zhang et al., 2014c; Khemka et al., 2016; Zhou et al., 2018). According to previous research, the lncRNAs discovered in this study differ from mRNAs in a variety of ways, including fewer exons, shorter transcript lengths, and lower conversation levels (Wang et al., 2019b; Yan et al., 2020). Although not uniformly distributed, the detected wheat lncRNAs were found to be scattered across all chromosomes. A similar tendency has been observed in other cereal crops such as rice and maize (Li et al., 2014b; Wang et al., 2018a).

The majority of the identified lncRNAs regulate the expression of genes associated with numerous biological processes by acting as target mimics or decoys of miRNA (Paraskevopoulou et al., 2013; Johnsson et al., 2014; Fan et al., 2018). Because lncRNAs also function through miRNAs for transcriptional, post-transcriptional, and epigenetic gene regulation through diverse molecular mechanisms, it is important to identify miRNAs that interact with lncRNA to find out their targets for having a better understanding of the disease responsiveness mechanism of plants. In our study, 199 lncRNAs were found to interact with 65 *T. aestivum* miRNAs, which targeted 757 distinct mRNA transcripts. Among them, many target genes regulated by lncRNAs have critical roles in wheat stripe rust resistance (**Table 2**). We observed that lncRNA TCONS_00079585 showed interactions with miRNA tae-miR164 (**Figure 4F**), which target genes encoding for putative disease resistance RPP13-like protein 3 and NAC domain protein. NAC transcription factor that serves as the target of tae-miR164 is similarly reported to be involved in wheat resistance to stripe rust (Feng et al., 2014a). Six lncRNAs (TCONS_00046493, TCONS_00142862, TCONS_00142861, TCONS_00142859, TCONS_00146761, and TCONS_00151741) showed interaction with miRNA tae-miR5175-5p (**Figure 4E**), which targets 20 monodehydroascorbate reductase genes, which were also previously reported to contribute to adult wheat plant resistance to stripe rust through ROS metabolism (Feng et al., 2014b). We found six lncRNAs (TCONS_00155902, TCONS_00103472, TCONS_00147277, TCONS_00147276, TCONS_00029083, and TCONS_00013986) acting as endogenous target mimics (eTMs) of miRNA tae-miR1127a (**Figure 4E**) that target 39 wheat mRNA sequences which encode for disease resistance-like or NBS-LRR proteins, NAC domain-containing protein 78, peroxidase 70-like protein, and monodehydroascorbate reductase, which are all related to stripe rust resistance. In the literature, tae-miR1127 is also mentioned to target serine/threonine-protein kinase protein (Sharma et al., 2020), lipoxygenase protein (An et al., 2016), and bidirectional sugar transporter SWEET9 protein (Huai et al., 2019, 20), which have a strong role in wheat stripe rust. Five lncRNAs (TCONS_00141524, TCONS_00141522, TCONS_00118627, TCONS_00019414, and TCONS_00019413), which act as eTMs of miRNA, tae-miR408, target a chemocyanin-like protein gene (*TaCLP1*), which play positive roles in wheat response to high salinity, heavy cupric stress, and stripe rust (Feng et al., 2013), and miRNA (tae-miR408) has been also identified in our study to target SUMO-activating enzyme subunit 1A. Several other miRNAs such as miR167, miR171, miR444, miR1129, and miR1138 were reported in the literature (Gupta et al., 2012) to play important roles in wheat rust resistance. We also confirmed lncRNAs acting as eTMs of tae-miR167, tae-miR171, tae-miR444, and their corresponding target genes involved in the wheat–*Pst* interactions. Another study showed that a number of the miRNAs such as miR2592s, miR869.1, and miR169b were highly differentially regulated, showing more than 200-fold change upon fungal inoculation (Inal et al., 2014). We also confirmed two conserved miRNAs and their corresponding target genes involved in the wheat–*Pst* interactions (Feng et al., 2013, 2014b). Most of the identified miRNAs mentioned above were predicted to be *Pst*-responsive miRNAs. Therefore, the identification of miRNAs and their targets will lay a comprehensive foundation for unraveling complex miRNA-mediated regulatory networks and their contribution to the wheat response to the *Pst* infection. The interaction of lncRNAs with miRNAs and mRNAs revealed that they play important roles in wheat stripe rust response, but further research is needed to confirm the precise significance of individual lncRNAs. The GO analysis of the target genes of the lncRNAs showed that some biological processes and molecular functions, such as metabolic, biological regulation, response to stimulus, localization, immune system process, binding, ATP-dependent activity, catalytic, antioxidant, and transporter activities, could be involved in response to stripe rust in wheat (Wang et al., 2021; Y et al., 2021). The annotations of the most significantly enriched KEGG pathways associated with the target genes of the identified lncRNAs are metabolism of ascorbate and aldarate, carbon fixation in photosynthetic organisms, metabolic pathways, arginine biosynthesis, purine metabolism, and the biosynthesis of secondary metabolites, which can play pivotal roles in the mechanism of disease response in plant (Erayman et al., 2015; Karki et al., 2021). Protein domains such as protein kinases, WRKY, and NAC domain are crucial in mounting an effective defensive response, which have been identified in the target genes of the lncRNAs (Afzal et al., 2008; Wang et al., 2009; Wang et al., 2020). Collectively, these observations suggest that lncRNAs play a pivotal part in the regulation of biotic stress tolerance in stripe rust infection in wheat.

## 5 Conclusion

Regulatory RNA like lncRNA plays an important role in different biological processes and metabolic activities in many plants by gene regulation, but their study in wheat (*T. aestivum*) due to biotic stresses is very limited, and to our knowledge, only few reports are available till now. This study focuses on the identification of lncRNAs and their expression in response to stripe rust pathogen attack in wheat near-isogenic lines susceptible and immune to *Pst*. In summary, the computational analysis allowed us to identify 6,807 lncRNAs in *T. aestivum*, and among them, 10 lncRNAs were differentially expressed between two NILs. A total of 199 lncRNAs act as target mimic of wheat miRNAs, which targets many genes that are involved in important defense processes against wheat stripe rust. The results provide useful information to further explore the activity of non-protein-coding genes in defense against stripe rust in wheat, and understanding the mechanism of gene regulation will contribute to the improvement of breeding programs for resistant wheat commercialization.

## Data availability statement

The datasets presented in this study can be found in online repositories. The names of the repository/repositories and accession number(s) can be found in the article/**Supplementary Material**. The datasets generated for this study can be found in the NCBI Sequence Read Archive (SRA) bioproject under accession numbers PRJNA613349.

## Author contributions

PD and MG analyzed the RNA-seq data and drafted the original manuscript. SG assisted in writing the draft. MG and DM conceptualized, investigated, and revised the manuscript. AS conducted the experiment and collected the samples at different time durations and reviewed the manuscript. SK, SB, and AR reviewed and edited the manuscript. BS and ZM carried out the wet lab validation. All authors contributed to the article and approved the submitted version.

## References

[B1] Afzal A. J. Wood A. J. Lightfoot D. A. (2008). Plant receptor-like serine threonine kinases: roles in signaling and plant defense. Mol. Plant Microbe Interact. 21, 507–517. doi: 10.1094/MPMI-21-5-0507 18393610

[B2] An T. Cai Y. Zhao S. Zhou J. Song B. Bux H.. (2016). Brachypodium distachyon T-DNA insertion lines: a model pathosystem to study nonhost resistance to wheat stripe rust. Sci. Rep. 6, 25510. doi: 10.1038/srep25510 27138687 PMC4853781

[B3] Aukerman M. J. Sakai H. (2003). Regulation of flowering time and floral organ identity by a microRNA and its APETALA2-like target genes. Plant Cell 15, 2730–2741. doi: 10.1105/tpc.016238 14555699 PMC280575

[B4] Bardou F. Ariel F. Simpson C. G. Romero-Barrios N. Laporte P. Balzergue S.. (2014). Long noncoding RNA modulates alternative splicing regulators in arabidopsis. Dev. Cell 30, 166–176. doi: 10.1016/j.devcel.2014.06.017 25073154

[B5] Berry S. Dean C. (2015). Environmental perception and epigenetic memory: mechanistic insight through FLC. Plant J. 83, 133–148. doi: 10.1111/tpj.12869 25929799 PMC4691321

[B6] Borgognone A. Sanseverino W. Aiese Cigliano R. Castanera R. (2019). Distribution, characteristics, and regulatory potential of long noncoding RNAs in brown-rot fungi. Int. J. Genomics 2019, e9702342. doi: 10.1155/2019/9702342 PMC6525899 31192251

[B7] Bu D. Luo H. Huo P. Wang Z. Zhang S. He Z.. (2021). KOBAS-i: intelligent prioritization and exploratory visualization of biological functions for gene enrichment analysis. Nucleic Acids Res. 49, W317–W325. doi: 10.1093/nar/gkab447 34086934 PMC8265193

[B8] Budak H. Kaya S. B. Cagirici H. B. (2020). Long non-coding RNA in plants in the era of reference sequences. Front. Plant Sci. 11. doi: 10.3389/fpls.2020.00276 PMC7080850 32226437

[B9] Chekanova J. A. (2015). Long non-coding RNAs and their functions in plants. Curr. Opin. Plant Biol. 27, 207–216. doi: 10.1016/j.pbi.2015.08.003 26342908

[B10] Chen X. (2005). Epidemiology and control of stripe rust [Puccinia striiformis f. sp. tritici] on wheat. Canadian Journal of Plant Pathology 27, 314–337. doi: 10.1080/07060660509507230

[B11] Chen X. (2012). Small RNAs in development – insights from plants. Curr. Opin. Genet. Dev. 22, 361–367. doi: 10.1016/j.gde.2012.04.004 22578318 PMC3419802

[B12] Chen Y.-E. Cui J.-M. Su Y.-Q. Yuan S. Yuan M. Zhang H.-Y. (2015). Influence of stripe rust infection on the photosynthetic characteristics and antioxidant system of susceptible and resistant wheat cultivars at the adult plant stage. Front. Plant Sci. 6. doi: 10.3389/fpls.2015.00779 PMC4585106 26442087

[B13] Chisholm S. T. Coaker G. Day B. Staskawicz B. J. (2006). Host-microbe interactions: shaping the evolution of the plant immune response. Cell 124, 803–814. doi: 10.1016/j.cell.2006.02.008 16497589

[B14] Conesa A. Götz S. García-Gómez J. M. Terol J. Talón M. Robles M. (2005). Blast2GO: a universal tool for annotation, visualization and analysis in functional genomics research. Bioinformatics 21, 3674–3676. doi: 10.1093/bioinformatics/bti610 16081474

[B15] Coram T. E. Settles M. L. Chen X. (2008). Transcriptome analysis of high-temperature adult-plant resistance conditioned by Yr39 during the wheat-Puccinia striiformis f. sp. tritici interaction. Mol. Plant Pathol. 9, 479–493. doi: 10.1111/j.1364-3703.2008.00476.x 18705862 PMC6640281

[B16] Dai X. Zhao P. X. (2011). psRNATarget: a plant small RNA target analysis server. Nucleic Acids Res. 39, W155–W159. doi: 10.1093/nar/gkr319 21622958 PMC3125753

[B17] Dangl J. L. Jones J. D. G. (2001). Plant pathogens and integrated defence responses to infection. Nature 411, 826–833. doi: 10.1038/35081161 11459065

[B18] Dhanoa J. K. Sethi R. S. Verma R. Arora J. S. Mukhopadhyay C. S. (2018). Long non-coding RNA: its evolutionary relics and biological implications in mammals: a review. J. Anim. Sci. Technol. 60, 25. doi: 10.1186/s40781-018-0183-7 30386629 PMC6201556

[B19] Dobon A. Bunting D. C. E. Cabrera-Quio L. E. Uauy C. Saunders D. G. O. (2016). The host-pathogen interaction between wheat and yellow rust induces temporally coordinated waves of gene expression. BMC Genomics 17, 380. doi: 10.1186/s12864-016-2684-4 27207100 PMC4875698

[B20] Dodds P. N. Rathjen J. P. (2010). Plant immunity: towards an integrated view of plant–pathogen interactions. Nat. Rev. Genet. 11, 539–548. doi: 10.1038/nrg2812 20585331

[B21] Du L. Zhang C. Liu Q. Zhang X. Yue B. (2018). Krait: an ultrafast tool for genome-wide survey of microsatellites and primer design. Bioinformatics 34, 681–683. doi: 10.1093/bioinformatics/btx665 29048524

[B22] Erayman M. Turktas M. Akdogan G. Gurkok T. Inal B. Ishakoglu E.. (2015). Transcriptome analysis of wheat inoculated with Fusarium graminearum. Front. Plant Sci. 6. doi: 10.3389/fpls.2015.00867 PMC4611148 26539199

[B23] Eulgem T. Weigman V. J. Chang H.-S. McDowell J. M. Holub E. B. Glazebrook J.. (2004). Gene expression signatures from three genetically separable resistance gene signaling pathways for downy mildew resistance. Plant Physiol. 135, 1129–1144. doi: 10.1104/pp.104.040444 15181204 PMC514145

[B24] Fan C.-N. Ma L. Liu N. (2018). Systematic analysis of lncRNA–miRNA–mRNA competing endogenous RNA network identifies four-lncRNA signature as a prognostic biomarker for breast cancer. J. Trans. Med. 16, 264. doi: 10.1186/s12967-018-1640-2 PMC6161429 30261893

[B25] Feng H. Duan X. Zhang Q. Li X. Wang B. Huang L.. (2014a). The target gene of tae-miR164, a novel NAC transcription factor from the NAM subfamily, negatively regulates resistance of wheat to stripe rust. Mol. Plant Pathol. 15, 284–296. doi: 10.1111/mpp.12089 24128392 PMC6638668

[B26] Feng H. Wang X. Zhang Q. Fu Y. Feng C. Wang B.. (2014b). Monodehydroascorbate reductase gene, regulated by the wheat PN-2013 miRNA, contributes to adult wheat plant resistance to stripe rust through ROS metabolism. Biochim. Biophys. Acta (BBA) - Gene Regul. Mech. 1839, 1–12. doi: 10.1016/j.bbagrm.2013.11.001 24269602

[B27] Feng H. Wang B. Zhang Q. Fu Y. Huang L. Wang X.. (2015). Exploration of microRNAs and their targets engaging in the resistance interaction between wheat and stripe rust. Front. Plant Sci. 6. doi: 10.3389/fpls.2015.00469 PMC4485317 26175740

[B28] Feng H. Zhang Q. Wang Q. Wang X. Liu J. Li M.. (2013). Target of tae-miR408, a chemocyanin-like protein gene (TaCLP1), plays positive roles in wheat response to high-salinity, heavy cupric stress and stripe rust. Plant Mol. Biol. 83, 433–443. doi: 10.1007/s11103-013-0101-9 23864359

[B29] Gao F. Cai Y. Kapranov P. Xu D. (2020). Reverse-genetics studies of lncRNAs—what we have learnt and paths forward. Genome Biol. 21, 93. doi: 10.1186/s13059-020-01994-5 32290841 PMC7155256

[B30] Griffiths-Jones S. Grocock R. J. van Dongen S. Bateman A. Enright A. J. (2006). miRBase: microRNA sequences, targets and gene nomenclature. Nucleic Acids Res. 34, D140–D144. doi: 10.1093/nar/gkj112 16381832 PMC1347474

[B31] Griffiths-Jones S. Saini H. K. van Dongen S. Enright A. J. (2008). miRBase: tools for microRNA genomics. Nucleic Acids Res. 36, D154–D158. doi: 10.1093/nar/gkm952 17991681 PMC2238936

[B32] Gruber A. R. Bernhart S. H. Lorenz R. (2015). “The viennaRNA web services,” in RNA Bioinformatics Methods in Molecular Biology. Ed. Picardi E. (New York, NY: Springer), 307–326. doi: 10.1007/978-1-4939-2291-8_19 25577387

[B33] Gupta O. P. Permar V. Koundal V. Singh U. D. Praveen S. (2012). MicroRNA regulated defense responses in Triticum aestivum L. during Puccinia graminis f.sp. tritici infection. Mol. Biol. Rep. 39, 817–824. doi: 10.1007/s11033-011-0803-5 21633895

[B34] Hao Z. Fan C. Cheng T. Su Y. Wei Q. Li G. (2015). Genome-wide identification, characterization and evolutionary analysis of long intergenic noncoding RNAs in cucumber. PloS One 10, e0121800. doi: 10.1371/journal.pone.0121800 25799544 PMC4370693

[B35] Heo J. B. Sung S. (2011). Vernalization-mediated epigenetic silencing by a long intronic noncoding RNA. Science 331, 76–79. doi: 10.1126/science.1197349 21127216

[B36] Huai B. Yang Q. Qian Y. Qian W. Kang Z. Liu J. (2019). ABA-induced sugar transporter taSTP6 promotes wheat susceptibility to stripe rust1 [OPEN]. Plant Physiol. 181, 1328–1343. doi: 10.1104/pp.19.00632 31540949 PMC6836835

[B37] Inal B. Türktaş M. Eren H. Ilhan E. Okay S. Atak M.. (2014). Genome-wide fungal stress responsive miRNA expression in wheat. Planta 240, 1287–1298. doi: 10.1007/s00425-014-2153-8 25156489

[B38] Isin M. Dalay N. (2015). LncRNAs and neoplasia. Clin. Chim. Acta 444, 280–288. doi: 10.1016/j.cca.2015.02.046 25748036

[B39] Jain N. Rani S. Sharma C. Sinha N. Singh A. Sharma J. B.. (2020). Large-scale stage-specific regulation of gene expression during host-pathogen interactions in CSP44 bread wheat carrying APR gene Lr48. Funct. Plant Biol. 47, 203–225. doi: 10.1071/FP18336 32007128

[B40] Jie Z. (2003) Histology and Ultrastructure of Incompatible Combination Between Puccinia striiformis and Wheat Cultivars with Resistance of Low Reaction Type. Available at: https://www.semanticscholar.org/paper/Histology-and-Ultrastructure-of-Incompatible-and-of-Jie/7334209fa6b990975f621f2a706357e9c1fefb4d (Accessed May 9, 2023).

[B41] Johnsson P. Lipovich L. Grandér D. Morris K. V. (2014). Evolutionary conservation of long non-coding RNAs; sequence, structure, function. Biochim. Biophys. Acta 1840, 1063–1071. doi: 10.1016/j.bbagen.2013.10.035 24184936 PMC3909678

[B42] Joshi R. K. Megha S. Basu U. Rahman M. H. Kav N. N. V. (2016). Genome Wide Identification and Functional Prediction of Long Non-Coding RNAs Responsive to Sclerotinia sclerotiorum Infection in Brassica napus. PloS One 11, e0158784. doi: 10.1371/journal.pone.0158784 27388760 PMC4936718

[B43] Kang S.-H. Sun Y.-D. Atallah O. O. Huguet-Tapia J. C. Noble J. D. Folimonova S. Y. (2019). A Long Non-Coding RNA of Citrus tristeza virus: Role in the Virus Interplay with the Host Immunity. Viruses 11, 436. doi: 10.3390/v11050436 31091710 PMC6563247

[B44] Kang Y.-J. Yang D.-C. Kong L. Hou M. Meng Y.-Q. Wei L.. (2017). CPC2: a fast and accurate coding potential calculator based on sequence intrinsic features. Nucleic Acids Res. 45, W12–W16. doi: 10.1093/nar/gkx428 28521017 PMC5793834

[B45] Karki K. Coolong T. Kousik C. Petkar A. Myers B. K. Hajihassani A.. (2021). The Transcriptomic Profile of Watermelon Is Affected by Zinc in the Presence of Fusarium oxysporum f. sp. niveum and Meloidogyne incognita. Pathogens 10, 796. doi: 10.3390/pathogens10070796 34201638 PMC8308719

[B46] Khemka N. Singh V. K. Garg R. Jain M. (2016). Genome-wide analysis of long intergenic non-coding RNAs in chickpea and their potential role in flower development. Sci. Rep. 6, 33297. doi: 10.1038/srep33297 27628568 PMC5024101

[B47] Kim E.-D. Sung S. (2012). Long noncoding RNA: unveiling hidden layer of gene regulatory networks. Trends Plant Sci. 17, 16–21. doi: 10.1016/j.tplants.2011.10.008 22104407

[B48] Kim D.-H. Sung S. (2017). Vernalization-triggered intragenic chromatin loop formation by long noncoding RNAs. Dev. Cell 40, 302–312.e4. doi: 10.1016/j.devcel.2016.12.021 28132848 PMC5303624

[B49] Kwenda S. Birch P. R. J. Moleleki L. N. (2016). Genome-wide identification of potato long intergenic noncoding RNAs responsive to Pectobacterium carotovorum subspecies brasiliense infection. BMC Genomics 17, 614. doi: 10.1186/s12864-016-2967-9 27515663 PMC4982125

[B50] Li L. Eichten S. R. Shimizu R. Petsch K. Yeh C.-T. Wu W.. (2014b). Genome-wide discovery and characterization of maize long non-coding RNAs. Genome Biol. 15, R40. doi: 10.1186/gb-2014-15-2-r40 24576388 PMC4053991

[B51] Li W. Li C. Li S. Peng M. (2017). Long noncoding RNAs that respond to Fusarium oxysporum infection in ‘Cavendish’ banana (Musa acuminata). Sci. Rep. 7, 16939. doi: 10.1038/s41598-017-17179-3 29209086 PMC5717134

[B52] Li S. Yamada M. Han X. Ohler U. Benfey P. N. (2016). High-resolution expression map of the arabidopsis root reveals alternative splicing and lincRNA regulation. Dev. Cell 39, 508–522. doi: 10.1016/j.devcel.2016.10.012 27840108 PMC5125536

[B53] Li A. Zhang J. Zhou Z. (2014a). PLEK: a tool for predicting long non-coding RNAs and messenger RNAs based on an improved k-mer scheme. BMC Bioinf. 15, 311. doi: 10.1186/1471-2105-15-311 PMC4177586 25239089

[B54] Liu J. Jung C. Xu J. Wang H. Deng S. Bernad L.. (2012). Genome-wide analysis uncovers regulation of long intergenic noncoding RNAs in Arabidopsis. Plant Cell 24, 4333–4345. doi: 10.1105/tpc.112.102855 23136377 PMC3531837

[B55] Liu H. Wang R. Mao B. Zhao B. Wang J. (2019). Identification of lncRNAs involved in rice ovule development and female gametophyte abortion by genome-wide screening and functional analysis. BMC Genomics 20, 90. doi: 10.1186/s12864-019-5442-6 30691391 PMC6348626

[B56] Liu B. Xue X. Cui S. Zhang X. Han Q. Zhu L.. (2010). Cloning and characterization of a wheat beta-1,3-glucanase gene induced by the stripe rust pathogen Puccinia striiformis f. sp. tritici. Mol. Biol. Rep. 37, 1045–1052. doi: 10.1007/s11033-009-9823-9 19757158

[B57] Matzke M. A. Mosher R. A. (2014). RNA-directed DNA methylation: an epigenetic pathway of increasing complexity. Nat. Rev. Genet. 15, 394–408. doi: 10.1038/nrg3683 24805120

[B58] Mercer T. R. Dinger M. E. Mattick J. S. (2009). Long non-coding RNAs: insights into functions. Nat. Rev. Genet. 10, 155–159. doi: 10.1038/nrg2521 19188922

[B59] Mir Z. A. Chauhan D. Pradhan A. Srivastava V. Sharma D. Budhlakoti N. (2023). Comparative transcriptome profiling of near isogenic lines PBW343 and FLW29 to unravel defense related genes and pathways contributing to stripe rust resistance in wheat. Funct Integr Genomics 23, 169. doi: 10.1007/s10142-023-01104-1 37209309

[B60] Misganaw A. Abera S. (2017). Genetic diversity assessment of Guzoita abyssinica using EST derived simple sequence repeats (SSRs) markers. AJPS 11, 79–85. doi: 10.5897/AJPS2016.1512

[B61] Muthusamy M. Uma S. Suthanthiram B. Saraswathi M. S. Chandrasekar A. (2019). Genome-wide identification of novel, long non-coding RNAs responsive to Mycosphaerella eumusae and Pratylenchus coffeae infections and their differential expression patterns in disease-resistant and sensitive banana cultivars. Plant Biotechnol. Rep. 13, 73–83. doi: 10.1007/s11816-018-00514-z

[B62] Paraskevopoulou M. D. Georgakilas G. Kostoulas N. Reczko M. Maragkakis M. Dalamagas T. M.. (2013). DIANA-LncBase: experimentally verified and computationally predicted microRNA targets on long non-coding RNAs. Nucleic Acids Res. 41, D239–D245. doi: 10.1093/nar/gks1246 23193281 PMC3531175

[B63] Peart J. R. Mestre P. Lu R. Malcuit I. Baulcombe D. C. (2005). NRG1, a CC-NB-LRR protein, together with N, a TIR-NB-LRR protein, mediates resistance against tobacco mosaic virus. Curr. Biol. 15, 968–973. doi: 10.1016/j.cub.2005.04.053 15916955

[B64] Pinto L. R. Oliveira K. M. Marconi T. Garcia A. a. F. Ulian E. C. De Souza A. P. (2006). Characterization of novel sugarcane expressed sequence tag microsatellites and their comparison with genomic SSRs. Plant Breeding 125, 378–384. doi: 10.1111/j.1439-0523.2006.01227.x

[B65] Prasad P. Savadi S. Bhardwaj S. C. Gangwar O. P. Kumar S. (2019). Rust pathogen effectors: perspectives in resistance breeding. Planta 250, 1–22. doi: 10.1007/s00425-019-03167-6 30980247

[B66] Reinhart B. J. Weinstein E. G. Rhoades M. W. Bartel B. Bartel D. P. (2002). MicroRNAs in plants. Genes Dev. 16, 1616–1626. doi: 10.1101/gad.1004402 12101121 PMC186362

[B67] Sairam R. K. Rao K. V. Srivastava G. C. (2002). Differential response of wheat genotypes to long term salinity stress in relation to oxidative stress, antioxidant activity and osmolyte concentration. Plant Sci. 163, 1037–1046. doi: 10.1016/S0168-9452(02)00278-9

[B68] Shannon P. Markiel A. Ozier O. Baliga N. S. Wang J. T. Ramage D.. (2003). Cytoscape: a software environment for integrated models of biomolecular interaction networks. Genome Res. 13, 2498–2504. doi: 10.1101/gr.1239303 14597658 PMC403769

[B69] Sharma P. Bawa P. Yadav B. Kaur P. Jindal S. Yadav I.. (2020). Physical mapping of an adult plant stripe rust resistance gene from Triticum monococcum. J. Plant Biochem. Biotechnol. 29, 47–55. doi: 10.1007/s13562-019-00511-5

[B70] Shendure J. (2008). The beginning of the end for microarrays? Nat. Methods 5, 585–587. doi: 10.1038/nmeth0708-585 18587314

[B71] Shumayla Sharma S. Taneja M. Tyagi S. Singh K. Upadhyay S. K. (2017). Survey of High Throughput RNA-Seq Data Reveals Potential Roles for lncRNAs during Development and Stress Response in Bread Wheat. Front. Plant Sci. 8. doi: 10.3389/fpls.2017.01019 PMC5465302 28649263

[B72] Tav C. Tempel S. Poligny L. Tahi F. (2016). miRNAFold: a web server for fast miRNA precursor prediction in genomes. Nucleic Acids Res. 44, W181–W184. doi: 10.1093/nar/gkw459 27242364 PMC4987958

[B73] Trapnell C. Pachter L. Salzberg S. L. (2009). TopHat: discovering splice junctions with RNA-Seq. Bioinformatics 25, 1105–1111. doi: 10.1093/bioinformatics/btp120 19289445 PMC2672628

[B74] Trapnell C. Roberts A. Goff L. Pertea G. Kim D. Kelley D. R.. (2012). Differential gene and transcript expression analysis of RNA-seq experiments with TopHat and Cufflinks. Nat. Protoc. 7, 562–578. doi: 10.1038/nprot.2012.016 22383036 PMC3334321

[B75] Trapnell C. Williams B. A. Pertea G. Mortazavi A. Kwan G. van Baren M. J.. (2010). Transcript assembly and quantification by RNA-Seq reveals unannotated transcripts and isoform switching during cell differentiation. Nat. Biotechnol. 28, 511–515. doi: 10.1038/nbt.1621 20436464 PMC3146043

[B76] Varshney D. Rawal H. C. Dubey H. Bandyopadhyay T. Bera B. Kumar P. M.. (2019). Tissue specific long non-coding RNAs are involved in aroma formation of black tea. Ind. Crops Products 133, 79–89. doi: 10.1016/j.indcrop.2019.03.020

[B77] Wang X. Basnayake B. M. V. S. Zhang H. Li G. Li W. Virk N.. (2009). The Arabidopsis ATAF1, a NAC transcription factor, is a negative regulator of defense responses against necrotrophic fungal and bacterial pathogens. Mol. Plant Microbe Interact. 22, 1227–1238. doi: 10.1094/MPMI-22-10-1227 19737096

[B78] Wang K. C. Chang H. Y. (2011). Molecular mechanisms of long noncoding RNAs. Mol. Cell 43, 904–914. doi: 10.1016/j.molcel.2011.08.018 21925379 PMC3199020

[B79] Wang H.-L. V. Chekanova J. A. (2017). Long noncoding RNAs in plants. Adv. Exp. Med. Biol. 1008, 133–154. doi: 10.1007/978-981-10-5203-3_5 28815539 PMC6689229

[B80] Wang Y. Fan X. Lin F. He G. Terzaghi W. Zhu D.. (2014). Arabidopsis noncoding RNA mediates control of photomorphogenesis by red light. Proc. Natl. Acad. Sci. U.S.A. 111, 10359–10364. doi: 10.1073/pnas.1409457111 24982146 PMC4104870

[B81] Wang G.-F. Fan R. Wang X. Wang D. Zhang X. (2015). TaRAR1 and TaSGT1 associate with TaHsp90 to function in bread wheat (Triticum aestivum L.) seedling growth and stripe rust resistance. Plant Mol. Biol. 87, 577–589. doi: 10.1007/s11103-015-0298-x 25697954

[B82] Wang A. Hu J. Gao C. Chen G. Wang B. Lin C.. (2019a). Genome-wide analysis of long non-coding RNAs unveils the regulatory roles in the heat tolerance of Chinese cabbage (Brassica rapa ssp.chinensis). Sci. Rep. 9, 5002. doi: 10.1038/s41598-019-41428-2 30899041 PMC6428831

[B83] Wang C.-F. Huang L.-L. Buchenauer H. Han Q.-M. Zhang H.-C. Kang Z.-S. (2007). Histochemical studies on the accumulation of reactive oxygen species (O2– and H2O2) in the incompatible and compatible interaction of wheat—Puccinia striiformis f. sp. tritici. Physiol. Mol. Plant Pathol. 71, 230–239. doi: 10.1016/j.pmpp.2008.02.006

[B84] Wang Z. Li B. Li Y. Zhai X. Dong Y. Deng M.. (2018d). Identification and characterization of long noncoding RNA in Paulownia tomentosa treated with methyl methane sulfonate. Physiol. Mol. Biol. Plants 24, 325–334. doi: 10.1007/s12298-018-0513-8 29515326 PMC5834995

[B85] Wang C.-Y. Liu S.-R. Zhang X.-Y. Ma Y.-J. Hu C.-G. Zhang J.-Z. (2017). Genome-wide screening and characterization of long non-coding RNAs involved in flowering development of trifoliate orange (Poncirus trifoliata L. Raf.). Sci. Rep. 7, 43226. doi: 10.1038/srep43226 28233798 PMC5324131

[B86] Wang Y. Luo X. Sun F. Hu J. Zha X. Su W.. (2018a). Overexpressing lncRNA LAIR increases grain yield and regulates neighbouring gene cluster expression in rice. Nat. Commun. 9, 3516. doi: 10.1038/s41467-018-05829-7 30158538 PMC6115402

[B87] Wang W. Wang J. Wei Q. Li B. Zhong X. Hu T.. (2019b). Transcriptome-Wide Identification and Characterization of Circular RNAs in Leaves of Chinese Cabbage (Brassica rapa L. ssp. pekinensis) in Response to Calcium Deficiency-Induced Tip-burn. Sci. Rep. 9, 14544. doi: 10.1038/s41598-019-51190-0 31601970 PMC6787205

[B88] Wang Y. Xu T. He W. Shen X. Zhao Q. Bai J.. (2018b). Genome-wide identification and characterization of putative lncRNAs in the diamondback moth, Plutella xylostella (L.). Genomics 110, 35–42. doi: 10.1016/j.ygeno.2017.08.003 28789862

[B89] Wang Y. Ye W. Wang Y. (2018c). Genome-wide identification of long non-coding RNAs suggests a potential association with effector gene transcription in Phytophthora sojae. Mol. Plant Pathol. 19, 2177–2186. doi: 10.1111/mpp.12692 29665235 PMC6638102

[B90] Wang H. Zou S. Li Y. Lin F. Tang D. (2020). An ankyrin-repeat and WRKY-domain-containing immune receptor confers stripe rust resistance in wheat. Nat. Commun. 11, 1353. doi: 10.1038/s41467-020-15139-6 32170056 PMC7070047

[B91] Wang Y. Huang L. Luo W. Jin Y. Gong F. He J.. (2021). Transcriptome analysis provides insights into the mechanisms underlying wheat cultivar Shumai126 responding to stripe rust. Gene 768, 145290. doi: 10.1016/j.gene.2020.145290 33157204

[B92] Xin M. Wang Y. Yao Y. Song N. Hu Z. Qin D.. (2011). Identification and characterization of wheat long non-protein coding RNAs responsive to powdery mildew infection and heat stress by using microarray analysis and SBS sequencing. BMC Plant Biol. 11, 61. doi: 10.1186/1471-2229-11-61 21473757 PMC3079642

[B93] Xing Q. Zhang W. Liu M. Li L. Li X. Yan J. (2019). Genome-wide identification of long non-coding RNAs responsive to lasiodiplodia theobromae infection in grapevine. Evolutionary Bioinf. 15, 117693431984136. doi: 10.1177/1176934319841362 PMC6449811 30992656

[B94] Yadav I. S. Sharma A. Kaur S. Nahar N. Bhardwaj S. C. Sharma T. R.. (2016). Comparative Temporal Transcriptome Profiling of Wheat near Isogenic Line Carrying Lr57 under Compatible and Incompatible Interactions. Front. Plant Sci. 7. doi: 10.3389/fpls.2016.01943 PMC5179980 28066494

[B95] Yan X. Ma L. Yang M. (2020). Identification and characterization of long non-coding RNA (lncRNA) in the developing seeds of Jatropha curcas. Sci. Rep. 10, 10395. doi: 10.1038/s41598-020-67410-x 32587349 PMC7316758

[B96] Ye J. Zhang Y. Cui H. Liu J. Wu Y. Cheng Y.. (2018). WEGO 2.0: a web tool for analyzing and plotting GO annotations 2018 update. Nucleic Acids Res. 46, W71–W75. doi: 10.1093/nar/gky400 29788377 PMC6030983

[B97] Y W. L H. W L. Y J. F G. J H. (2021). Transcriptome analysis provides insights into the mechanisms underlying wheat cultivar Shumai126 responding to stripe rust. Gene 768. doi: 10.1016/j.gene.2020.145290 33157204

[B98] Yu Y. Jia T. Chen X. (2017). The ‘how’ and ‘where’ of plant microRNAs. New Phytol. 216, 1002–1017. doi: 10.1111/nph.14834 29048752 PMC6040672

[B99] Yu X. Wang X. Wang C. Chen X. Qu Z. Yu X.. (2010). Wheat defense genes in fungal (Puccinia striiformis) infection. Funct. Integr. Genomics 10, 227–239. doi: 10.1007/s10142-010-0161-8 20186453

[B100] Zhang H. Chen X. Wang C. Xu Z. Wang Y. Liu X.. (2013a). Long non-coding genes implicated in response to stripe rust pathogen stress in wheat (Triticum aestivum L.). Mol. Biol. Rep. 40, 6245–6253. doi: 10.1007/s11033-013-2736-7 24065539

[B101] Zhang W. Han Z. Guo Q. Liu Y. Zheng Y. Wu F.. (2014b). Identification of maize long non-coding RNAs responsive to drought stress. PloS One 9, e98958. doi: 10.1371/journal.pone.0098958 24892290 PMC4044008

[B102] Zhang H. Hu Y. Wang C. Ji W. (2011). Gene expression in wheat induced by inoculation with Puccinia striiformis west. Plant Mol. Biol. Rep. 29, 458–465. doi: 10.1007/s11105-010-0245-6

[B103] Zhang H. Hu Y. Yang B. Xue F. Wang C. Kang Z.. (2013b). Isolation and characterization of a wheat IF2 homolog required for innate immunity to stripe rust. Plant Cell Rep. 32, 591–600. doi: 10.1007/s00299-013-1390-9 23397275

[B104] Zhang Y.-C. Liao J.-Y. Li Z.-Y. Yu Y. Zhang J.-P. Li Q.-F.. (2014c). Genome-wide screening and functional analysis identify a large number of long noncoding RNAs involved in the sexual reproduction of rice. Genome Biol. 15, 512. doi: 10.1186/s13059-014-0512-1 25517485 PMC4253996

[B105] Zhang J. Yang Z. Feng P. Zhong X. Ma Q. Su Q.. (2019). Identification and the potential roles of long non-coding RNAs in cotton leaves damaged by Aphis gossypii. Plant Growth Regul. 88, 215–225. doi: 10.1007/s10725-019-00500-7

[B106] Zhang H. Yang Y. Wang C. Liu M. Li H. Fu Y.. (2014a). Large-scale transcriptome comparison reveals distinct gene activations in wheat responding to stripe rust and powdery mildew. BMC Genomics 15, 898. doi: 10.1186/1471-2164-15-898 25318379 PMC4201691

[B107] Zhou Y. Cho W. K. Byun H.-S. Chavan V. Kil E.-J. Lee S.. (2019). Genome-wide identification of long non-coding RNAs in tomato plants irradiated by neutrons followed by infection with Tomato yellow leaf curl virus. PeerJ 7, e6286. doi: 10.7717/peerj.6286 30713817 PMC6354667

[B108] Zhou W. Shi H. Wang Z. Zhao Y. Gou X. Li C.. (2020). Identification of lncRNAs involved in wheat tillering development in two pairs of near-isogenic lines. Funct. Integr. Genomics 20, 669–679. doi: 10.1007/s10142-020-00742-z 32488459

[B109] Zhou R. Zhu Y. Zhao J. Fang Z. Wang S. Yin J.. (2018). Transcriptome-Wide Identification and Characterization of Potato Circular RNAs in Response to Pectobacterium carotovorum Subspecies brasiliense Infection. Int. J. Mol. Sci. 19, 71. doi: 10.3390/ijms19010071 PMC5796021 29280973

