# Supplementary material for: Genome-wide identification and characterization of Puccinia striiformis-responsive lncRNAs in Triticum aestivum
Source: Front Plant Sci. 2023 Aug 15;14:1120898. doi: 10.3389/fpls.2023.1120898 (PMC10465180; doi:10.3389/fpls.2023.1120898)
Supplement: Supplementary file 2 [file DataSheet_2.docx]

Supplementary Material

# Supplementary Figures and Tables

## Supplementary Figures


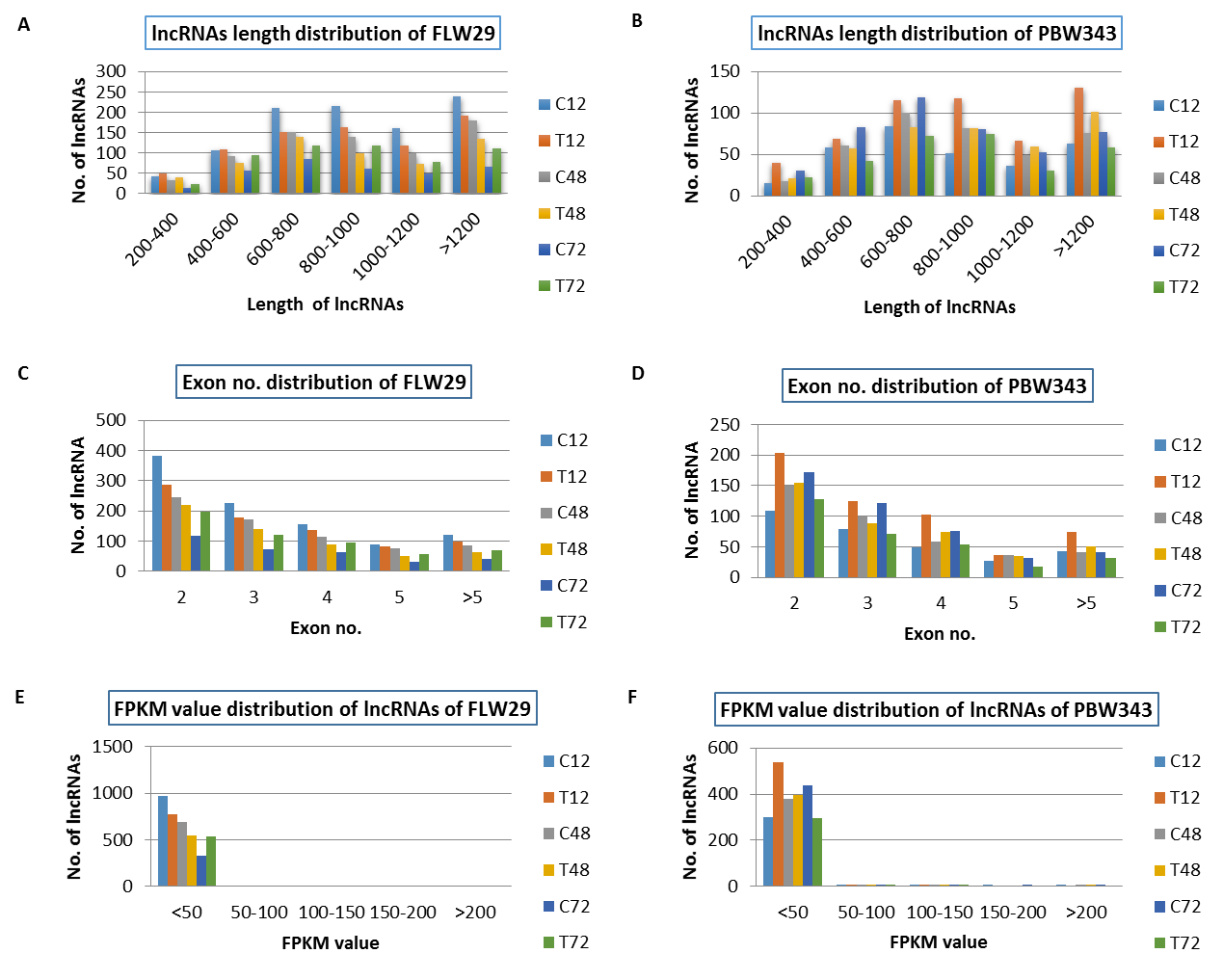


**Figure S1.** Basic Characterization of lncRNAs **(A)** Graphical representation of distribution of lncRNAs based on their length under different conditions of FLW29. **(B)** Graphical representation of distribution of lncRNAs based on their length under different conditions of PBW343 (**C)** Graphical representation of distribution of lncRNAs based on their exon number under different conditions of FLW29. **(D)** Graphical representation of distribution of lncRNAs based on their exon number under different conditions of PBW343. (**E)** Graphical representation of distribution of lncRNAs based on their FPLM value under different conditions of FLW29. **(F)** Graphical representation of distribution of lncRNAs based on their FPKM value under different conditions of PBW343.


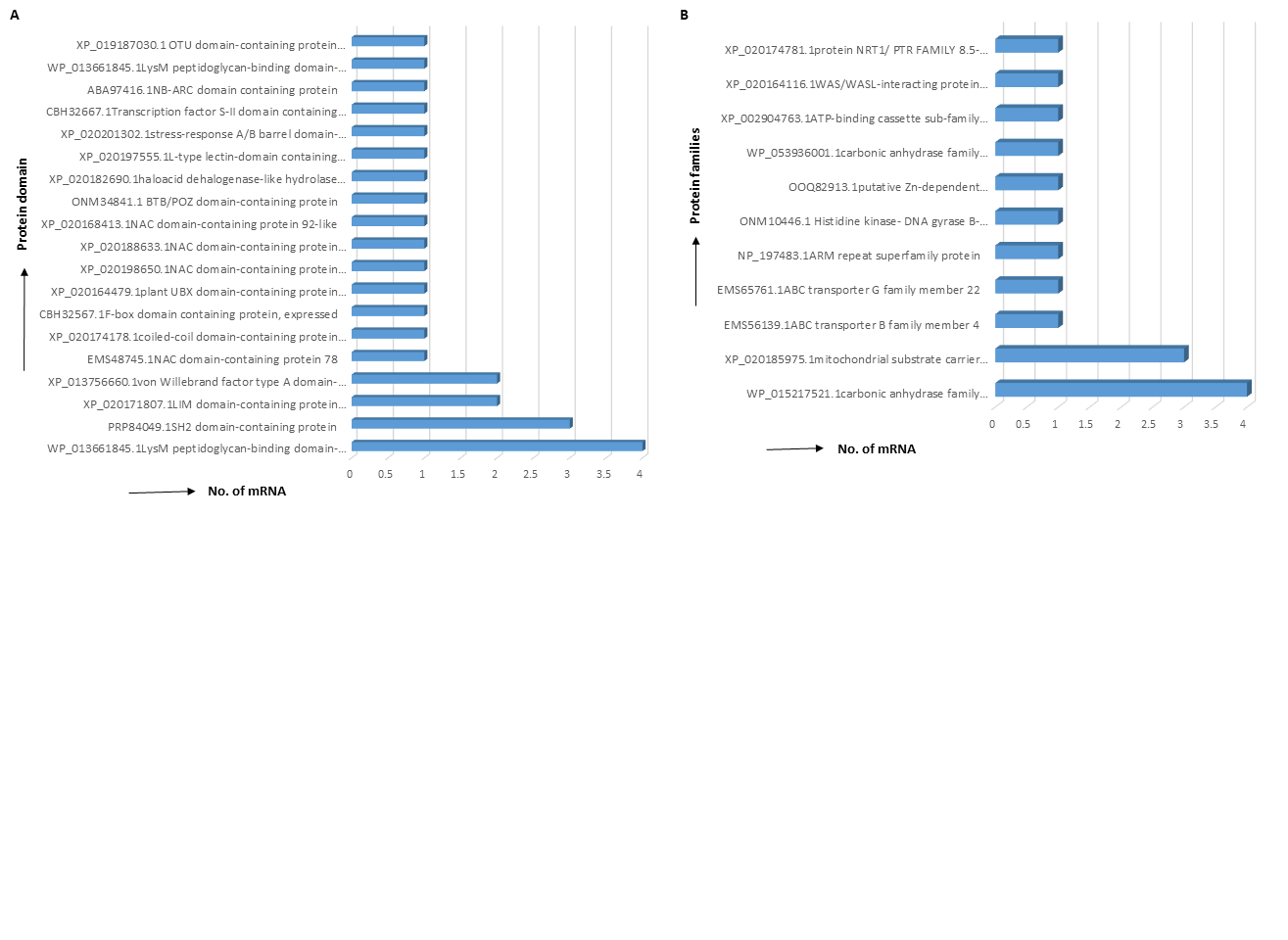


**Figure S2.** Protein domain and families associated with the mRNAs targets of the identified lncRNAs and their. **(A)** Protein domains related to the target genes of lncRNAs. **(B)** Protein families related to the target genes of lncRNAs.

**Figure S3.** Piechart depicting the type of SSRs (mono, di, tri, tetra, penta and hexa) identified from the lncRNAs.

## Supplementary Tables

**Table S1. Summary of data output quality generated by RNA-seq library**

| **Sample_name** | **Total_length(bp)** | **Raw_Reads** | **Clean reads** |  | **Q20** | **GC content** |
| --- | --- | --- | --- | --- | --- | --- |
| FLW29_C12_1 | 3209739956 | 21256556 | 17213957 |  | 97.11 | 56 |
| FLW29_C12_2 | 3214203516 | 21286116 | 16405018 |  | 97.08 | 55 |
| FLW29_C12_3 | 4977881850 | 33185879 | 20809439 |  | 96.56 | 55 |
| FLW29_T12_1 | 3843817344 | 25455744 | 21540764 |  | 97.17 | 52 |
| FLW29_T12_2 | 3532328051 | 23392901 | 18854033 |  | 97.08 | 55 |
| FLW29_T12_3 | 4125667050 | 27504447 | 22675040 |  | 96.58 | 54 |
| FLW29_C48_1 | 2858678697 | 18931647 | 14046884 |  | 97.25 | 54 |
| FLW29_C48_2 | 3228697704 | 21382104 | 15372485 |  | 96.54 | 52 |
| FLW29_C48_3 | 3517247250 | 23448315 | 13586886 |  | 96.71 | 55 |
| FLW29_T48_1 | 2819788496 | 18674096 | 15751323 |  | 96.8 | 55 |
| FLW29_T48_2 | 3038268886 | 20120986 | 14953229 |  | 94.85 | 54 |
| FLW29_T48_3 | 3630921900 | 24206146 | 18905493 |  | 97.79 | 57 |
| FLW29_C72_1 | 4691275500 | 31275170 | 12335040 |  | 96.93 | 55 |
| FLW29_C72_2 | 3271770150 | 21811801 | 13936157 |  | 96.75 | 57 |
| FLW29_C72_3 | 3438044550 | 22920297 | 15609376 |  | 96.56 | 59 |
| FLW29_T72_1 | 3814890300 | 25432602 | 16759359 |  | 97.29 | 58 |
| FLW29_T72_2 | 3824763300 | 25498422 | 16037136 |  | 95.33 | 57 |
| FLW29_T72_3 | 3697082250 | 24647215 | 15618145 |  | 96.93 | 57 |
| PBW343_C12_1 | 3793209996 | 25120596 | 19321511 |  | 97.03 | 53 |
| PBW343_C12_2 | 3437461999 | 22764649 | 18867750 |  | 96.75 | 55 |
| PBW343_C12_3 | 3070751850 | 20471679 | 12221662 |  | 96.8 | 58 |
| PBW343_T12_1 | 3609344393 | 23902943 | 18753237 |  | 97.12 | 55 |
| PBW343_T12_2 | 2576179290 | 17060790 | 13728156 |  | 97.26 | 55 |
| PBW343_T12_3 | 4523106900 | 30154046 | 21423482 |  | 97.12 | 57 |
| PBW343_C48_1 | 2810047637 | 18609587 | 11893910 |  | 97.39 | 55 |
| PBW343_C48_2 | 2025913546 | 13416646 | 9396276 |  | 97.75 | 53 |
| PBW343_C48_3 | 3055026900 | 20366846 | 15837614 |  | 97.45 | 55 |
| PBW343_T48_1 | 3413179236 | 22603836 | 16962679 |  | 96.81 | 53 |
| PBW343_T48_2 | 1137479527 | 7532977 | 5856688 |  | 97.39 | 55 |
| PBW343_T48_3 | 4709403000 | 31396020 | 25185317 |  | 96.9 | 57 |
| PBW343_C72_1 | 4703747100 | 31358314 | 23596443 |  | 96.93 | 57 |
| PBW343_C72_2 | 4409618700 | 29397458 | 19497104 |  | 96.9 | 56 |
| PBW343_C72_3 | 3677173650 | 24514491 | 16588666 |  | 96.75 | 58 |
| PBW343_T72_1 | 4715553600 | 31437024 | 22416796 |  | 96.81 | 57 |
| PBW343_T72_2 | 4549023300 | 30326822 | 18912976 |  | 95.64 | 59 |
| PBW343_T72_3 | 4298551050 | 28657007 | 20853807 |  | 96.37 | 59 |

**Table S2. Summary statistics of alignment reads of FLW29 and PBW343 over the reference genome**

| **Time interval** | | **Condition** | **Left read alignment** | | **Right read alignment** | | **Overall alignment** |
| --- | --- | --- | --- | --- | --- | --- | --- |
|  |  |  | **Mapped once** | **Multiple times** | **Mapped once** | **Multiple times** |  |
| **FLW29** | | | | | | | |
| 12 hours | Control | | 31649239 (76.0%) | 6363035 (20.1%) | 31349783 (75.3%) | 6300088 (20.1%) | 75.60% |
|  | Treatment | | 53267279 (82.7%) | 15196935 (28.5%) | 52930531 (82.2%) | 15113185 (28.6%) | 82.50% |
| 48 hours | Control | | 30356419 (81.8%) | 6501309 (21.4%) | 30070325 (81.0%) | 6437464 (21.4%) | 81.40% |
|  | Treatment | | 40026787 (83.4%) | 9025148 (22.5%) | 39502110 (82.3%) | 8898411 (22.5%) | 82.80% |
| 72 hours | Control | | 49151594 (82.4%) | 12788519 (26.0%) | 48554467 (81.4%) | 12583362 (25.9%) | 81.90% |
|  | Treatment | | 51171403 (82.3%) | 23656262 (46.2%) | 50697391 (81.5%) | 23440860 (46.2%) | 81.90% |
| **PBW343** | | | | | | | |
| 12 hours | Control | | 46113870 (81.60%) | 8688415 (18.8%) | 45448607 (80.4%) | 8539387 (18.8%) | 81.00% |
|  | Treatment | | 50180817 (83.2%) | 9876127 (19.7%) | 49517757 (82.1%) | 9726061 (19.6%) | 82.70% |
| 48 hours | Control | | 34195797 (79.5%) | 6560202 (19.2%) | 33907377 (78.8%) | 6515259 (19.2%) | 79.20% |
|  | Treatment | | 40783766 (82.2%) | 6498902 (15.9%) | 40242936 (81.1%) | 6406799 (15.9%) | 81.70% |
| 72 hours | Control | | 32560160 (77.7%) | 8639096 (26.5%) | 32154255 (76.8% | 8521343 (26.5%) | 77.30% |
|  | Treatment | | 34278421 (70.8%) | 11066710 (32.3%) | 34034197 (70.3%) | 10973159 (32.2%) | 70.50% |

**Table S3** List of samples under different conditions

| **Hours After Inoculation** | **Variety** | | |  |
| --- | --- | --- | --- | --- |
|  | **PBW343** | | **FLW29** | |
|  | Control | Treatment | Control | Treatment |
| 12 hours | C12-R1 | T12-R1 | C12-R1 | T12-R1 |
|  | C12-R2 | T12-R2 | C12-R2 | T12-R2 |
|  | C12-R3 | T12-R3 | C12-R3 | T12-R3 |
| 48 hours | C48-R1 | T48-R1 | C48-R1 | T48-R1 |
|  | C48-R2 | T48-R2 | C48-R2 | T48-R2 |
|  | C48-R3 | T48-R3 | C48-R3 | T48-R3 |
| 72 hours | C72-R1 | T72-R1 | C12-R1 | T72-R1 |
|  | C72-R2 | T72-R2 | C12-R2 | T72-R2 |
|  | C72-R3 | T72-R3 | C12-R3 | T72-R3 |

**Table S4(A):** Lengthwise characterization of lncRNAs of FLW29 line under different conditions

| **Length of lncRNAs** | **12 hours** | | **48 hours** | | **72 hours** | |
| --- | --- | --- | --- | --- | --- | --- |
| **(No. of nucleotides)** | **Control** | **Treated** | **Control** | **Treated** | **Control** | **Treated** |
| Minimum | 225 | 203 | 219 | 223 | 204 | 236 |
| Maximum | 3886 | 4460 | 3424 | 2953 | 4399 | 3394 |
| Average | 1007 | 973 | 986 | 966 | 938 | 926 |

**Table S4(B) :** Lengthwise characterization of lncRNAs of PBW343 line under different conditions.

| **Length of lncRNAs** | **12 hours** | | **48 hours** | | **72 hours** | |
| --- | --- | --- | --- | --- | --- | --- |
| **(No. of nucleotides)** | **Control** | **Treated** | **Control** | **Treated** | **Control** | **Treated** |
| Minimum | 203 | 223 | 204 | 219 | 214 | 214 |
| Maximum | 4379 | 4138 | 2666 | 4045 | 2666 | 2963 |
| Average | 911 | 968 | 911 | 984 | 874 | 902 |

**Table S4(C):** Distribution of lncRNAs of FLW29 based on their length under different conditions

| **Length of lncRNAs** | **12 hours** | | **48 hours** | | **72 hours** | |
| --- | --- | --- | --- | --- | --- | --- |
|  | **Control** | **Treated** | **Control** | **Treated** | **Control** | **Treated** |
| 200-400 | 43 | 48 | 32 | 40 | 13 | 23 |
| 400-600 | 107 | 108 | 92 | 74 | 57 | 94 |
| 600-800 | 210 | 152 | 152 | 140 | 84 | 117 |
| 800-1000 | 215 | 162 | 140 | 100 | 60 | 118 |
| 1000-1200 | 161 | 117 | 100 | 72 | 49 | 78 |
| >1200 | 240 | 192 | 180 | 134 | 66 | 110 |

**Table S4(D):** Distribution of lncRNAs of PBW343 based on their length line under different conditions.

| **Length of lncRNAs** | **12 hours** | | **48 hours** | | **72 hours** | |
| --- | --- | --- | --- | --- | --- | --- |
|  | **Control** | **Treated** | **Control** | **Treated** | **Control** | **Treated** |
| 200-400 | 15 | 40 | 18 | 21 | 30 | 22 |
| 400-600 | 58 | 69 | 61 | 57 | 83 | 42 |
| 600-800 | 84 | 116 | 100 | 83 | 119 | 73 |
| 800-1000 | 51 | 118 | 82 | 82 | 80 | 75 |
| 1000-1200 | 36 | 67 | 49 | 60 | 53 | 31 |
| >1200 | 63 | 131 | 76 | 101 | 77 | 59 |

**Table S5(A):** Distribution of lncRNAs of Flw29 line based on their exon count under different conditions.

| **Exon count of lncRNAs** | **12 hours** | | **48 hours** | | **72 hours** | |
| --- | --- | --- | --- | --- | --- | --- |
| 2 | 384 | 288 | 246 | 221 | 117 | 199 |
| 3 | 226 | 177 | 171 | 139 | 74 | 122 |
| 4 | 157 | 136 | 116 | 88 | 64 | 94 |
| 5 | 90 | 81 | 77 | 50 | 33 | 56 |
| >5 | 120 | 97 | 86 | 62 | 41 | 69 |

**Table S5(B):** Distribution of lncRNAs of Pbw343 line based on their exon count under different conditions.

| **Exon count of lncRNAs** | **12 hours** | | **48 hours** | | **72 hours** | |
| --- | --- | --- | --- | --- | --- | --- |
|  | **Control** | **Treated** | **Control** | **Treated** | **Control** | **Treated** |
| 2 | 109 | 204 | 150 | 155 | 172 | 128 |
| 3 | 79 | 125 | 100 | 89 | 122 | 71 |
| 4 | 49 | 103 | 58 | 75 | 76 | 54 |
| 5 | 27 | 36 | 37 | 35 | 31 | 17 |
| >5 | 43 | 74 | 41 | 50 | 41 | 32 |

**Table S6(A):** Distribution of lncRNAs of FLW29 line based on their FPKM value under different conditions.

| **Length of lncRNAs** | **12 hours** | | **48 hours** | | **72 hours** | |
| --- | --- | --- | --- | --- | --- | --- |
| <50 | 972 | 776 | 690 | 551 | 326 | 533 |
| 50-100 | 4 | 1 | 3 | 6 | 2 | 2 |
| 100-200 | 2 | 2 | 3 | 2 | 2 | 2 |
| >200 | 0 | 0 | 0 | 1 | 0 | 3 |

**Table S6(B):** Distribution of lncRNAs of PBW343 line based on their FPKM value under different conditions.

| **Length of lncRNAs** | **12 hours** | | **48 hours** | | **72 hours** | |
| --- | --- | --- | --- | --- | --- | --- |
|  | **Control** | **Treated** | **Control** | **Treated** | **Control** | **Treated** |
| <50 | 299 | 537 | 380 | 397 | 436 | 296 |
| 50-100 | 2 | 3 | 3 | 2 | 1 | 4 |
| 100-200 | 4 | 1 | 1 | 3 | 4 | 2 |
| >200 | 2 | 0 | 2 | 2 | 1 | 0 |

**Table S7(A):** Numbers of de-lncRNAs between Flw29 and PBW343 lines under both control and stress conditions at different time points.

| **Conditions** | **Upregulated** | **Downregulated** |
| --- | --- | --- |
| C12 | 0 | 1 |
| C48 | 1 | 0 |
| C72 | 4 | 0 |
|  |  |  |
| T12 | 0 | 3 |
| T48 | 1 | 1 |
| T72 | 2 | 0 |

**Table S7(B):** List of upregulated differentially expressed lncRNAs between Flw29 and PBW343 lines under both control and stress conditions.

| lncRNA_id | gene_id | Locus_id | Pbw_Expression_value | Flw_Expression_value | log2(fold_change) |
| --- | --- | --- | --- | --- | --- |
| TCONS_00076516 | XLOC_041001 | LS992089.1:620612954-620614697 | 1.64737 | 24.7918 | 3.91163 |
| TCONS_00093548 | XLOC_050123 | LS992092.1:382777382-382778534 | 16.2814 | 135.058 | 3.05229 |
| TCONS_00100461 | XLOC_054062 | LS992093.1:49531099-49531877 | 20.46 | 349.142 | 4.09293 |
| TCONS_00163170 | XLOC_087731 | LS992101.1:194669909-194673455 | 1.30631 | 20.9422 | 4.00284 |
| TCONS_00073476 | XLOC_039682 | LS992089.1:615109131-615109739 | 22.6046 | 357.537 | 3.9834 |

**Table S7(C):** List of downregulated differentially expressed lncRNAs between Flw29 and PBW343 lines under both control and stress conditions.

| lncRNA_id | gene_id | Locus_id | Pbw_Expression_value | Flw_Expression_value | log2(fold_change) |
| --- | --- | --- | --- | --- | --- |
| TCONS_00025410 | XLOC_013508 | LS992083.1:707222022-707236315 | 61.8343 | 8.08754 | -2.93463 |
| TCONS_00040012 | XLOC_021665 | LS992085.1:600086388-600087376 | 33.4476 | 2.80494 | -3.57586 |
| TCONS_00053873 | XLOC_029413 | LS992087.1:6088916-6090174 | 25.0546 | 3.03619 | -3.04474 |
| TCONS_00066365 | XLOC_035701 | LS992088.1:597736824-597737634 | 57.2792 | 4.5867 | -3.64248 |
| TCONS_00115070 | XLOC_061361 | LS992094.1:445645016-445654513 | 20.194 | 1.10241 | -4.19519 |

**Table S8:** Blast result of lncRNAs with the CDS sequences of wheat (sample)

| **query_id** | **subject_id** | **pct_identity** | **q_coverage** | **aln_length** | **e_value** | **Description/Function** |
| --- | --- | --- | --- | --- | --- | --- |
| TCONS_00009015 | TraesCS1B02G098700.1 | 99.587 | 100 | 242 | 8.65E-123 | Non-specific serine/threonine protein kinase |
| TCONS_00009015 | TraesCS1B02G098700.2 | 99.587 | 100 | 242 | 8.65E-123 | Non-specific serine/threonine protein kinase |
| TCONS_00009015 | TraesCS1A02G080700.1 | 97.934 | 100 | 242 | 4.05E-116 | Non-specific serine/threonine protein kinase |
| TCONS_00009015 | TraesCS1D02G082600.1 | 97.917 | 99.17355 | 240 | 5.24E-115 | Non-specific serine/threonine protein kinase |
| TCONS_00009015 | TraesCS1D02G082600.2 | 97.917 | 99.17355 | 240 | 5.24E-115 | Non-specific serine/threonine protein kinase |
| TCONS_00009015 | TraesCS1B02G098600.1 | 93.388 | 100 | 242 | 8.96E-98 | Non-specific serine/threonine protein kinase |
| TCONS_00009015 | TraesCS1D02G082500.3 | 93.388 | 100 | 242 | 8.96E-98 | Non-specific serine/threonine protein kinase |
| TCONS_00009015 | TraesCS1D02G082500.1 | 93.388 | 100 | 242 | 8.96E-98 | Non-specific serine/threonine protein kinase |
| TCONS_00009015 | TraesCS1D02G082200.1 | 92.562 | 100 | 242 | 1.94E-94 | Non-specific serine/threonine protein kinase |
| TCONS_00009015 | TraesCS1A02G080600.1 | 92.149 | 100 | 242 | 9.03E-93 | Non-specific serine/threonine protein kinase |
| TCONS_00009015 | TraesCS1A02G080500.2 | 90.909 | 100 | 242 | 9.09E-88 | Non-specific serine/threonine protein kinase |

**Table S9:** List of 15 significant blast hits of identified lncRNAs with other lncRNA databases of cereal.

| **Query_id** | **Subject_id** | **Percent identity** | **Coverage** | **Align-length** | **E value** | **Organism** |
| --- | --- | --- | --- | --- | --- | --- |
|  |  |  |  |  |  |  |
| TCONS_00015383 | CNT20165270 | 81.408 | 106.1303 | 554 | 3.82E-117 | *H. vulgare* |
| TCONS_00015383 | CNT20165269 | 81.408 | 106.1303 | 554 | 3.82E-117 | *H. vulgare* |
| TCONS_00138899 | CNT20164688 | 78.07 | 101.9678 | 570 | 2.57E-84 | *H. vulgare* |
| TCONS_00146772 | CNT20163366 | 88.112 | 101.0601 | 286 | 7.43E-92 | *H. vulgare* |
| TCONS_00146772 | CNT20168454 | 86.713 | 101.0601 | 286 | 3.48E-85 | *H. vulgare* |
| TCONS_00103782 | CNT20161355 | 91.304 | 100.2421 | 414 | 1.69E-159 | *H. vulgare* |
| TCONS_00034276 | CNT20165335 | 86.127 | 100.1931 | 519 | 7.75E-154 | *H. vulgare* |
| TCONS_00009343 | CNT20161858 | 86.606 | 100.1838 | 545 | 6.21E-165 | *H. vulgare* |
| TCONS_00032292 | lcl\|Osativa_LOC_Os07g41340.1 | 84.255 | 99.48187 | 384 | 7.25E-163 | *O. sativa* |
| TCONS_00133933 | lcl\|Osativa_LOC_Os02g57830.2 | 84.615 | 98.30028 | 347 | 3.34E-101 | *O. sativa* |
| TCONS_00000980 | lcl\|Osativa_LOC_Os10g33855.2 | 72.429 | 97.1519 | 614 | 2.06E-150 | *O. sativa* |
| TCONS_00006668 | lcl\|Zmays_GRMZM2G087549_T01 | 85.211 | 96.9697 | 512 | 1.02E-152 | *Zea mays* |
| TCONS_00146041 | lcl\|Sbicolor_Sobic.010G121300.1 | 79.531 | 95.79082 | 751 | 5.41E-167 | *Sorghum bicolor* |
| TCONS_00146041 | lcl\|Zmays_GRMZM2G099745_T01 | 77.644 | 94.51531 | 741 | 5.49E-157 | *Zea mays* |
| TCONS_00056119 | lcl\|Osativa_LOC_Os01g55020.2 | 82.09 | 93.48659 | 488 | 1.78E-115 | *O. sativa* |

**Table S15:** lncRNA primer details used in semiquantitative PCR analysis

| S.no | LncRNA IDs | Primer | Sequence |
| --- | --- | --- | --- |
| 1 | >TCONS_00093548 | Forward | 5’GAACGAGGGACTACTGGACTAT3’ |
|  |  | Reverse | 5’GAGAAGAAGGACCTGCAACTAC3’ |
| 2 | >TCONS_00163170 | Forward | 5’GGAGTTCTCAGCCCAATCAA3’ |
|  |  | Reverse | 5’GCCTCGTGTTATTGAGCAATTC3’ |
| 3 | >TCONS_00073476 | Forward | 5’GGTCGCCAATCCAAGTAGAA3’ |
|  |  | Reverse | 5’AGCAGAAGGAGGAGGATGA3’ |
| 4 | >TCONS_00025410 | Forward | 5’ACTCTTCTTGGTTCAGGGTTT3’ |
|  |  | Reverse | 5’CCGCTAGGATCGGAAATCTTAT3’ |
| 5 | >TCONS_00040012 | Forward | 5’CCCACGACCTCAACTATACAAG3’ |
|  |  | Reverse | 5’CCCTCTCGTTGCTATGTATCAC3’ |
| 6 | >TCONS_00053873 | Forward | 5’ACTCCATGGGCTACTAAATTCC3’ |
|  |  | Reverse | 5’ACCGTTCTGCAGCCATAAA3’ |
| 7 | >TCONS_00066365 | Forward | 5’CCGGGAACCTGAATGAGTTAG3’ |
|  |  | Reverse | 5’TGCTCCGTGGCGTTTATT3’ |
| 8 | >TCONS_00115070 | Forward | 5’GCACCCAAAGCCAACAATC3’ |
|  |  | Reverse | 5’GTCATCATCCAGGCTCCAATAA3’ |
